# Synthesis of zinc oxide nanoparticles using *Trichoderma harzianum* and its bio-efficacy on *Alternaria brassicae*

**DOI:** 10.3389/fmicb.2025.1506695

**Published:** 2025-02-13

**Authors:** Deep Narayan Mishra, Lakshman Prasad, Usha Suyal

**Affiliations:** Division of Plant Pathology, ICAR-Indian Agricultural Research Institute, New Delhi, India

**Keywords:** mustard, *Alternaria brassicae*, *Trichoderma*, M-ZnO NPs, ROS, antioxidant enzymes, *in vitro*

## Abstract

Increasing concerns about chemical fungicides require sustainable alternatives for crop protection. Microbe-mediated synthesis of metal nanoparticles offers a sustainable, eco-friendly and highly effective strategy for plant disease management. This study investigates the mycosynthesis of zinc oxide nanoparticles (ZnO NPs) using the culture filtrate of *Trichoderma harzianum* and their antifungal activity against *Alternaria brassicae*. Nanoparticles were synthesized under optimized conditions of cell-free culture filtrate (CFCF) concentration, substrate concentration, pH and temperature. Ultraviolet-visible (UV-Vis) spectroscopy confirmed an absorption peak between 200 and 400 nm, while X-ray diffraction (XRD) confirms the hexagonal crystal structure with an average size of 29 nm. Dynamic light scattering (DLS) and zeta potential analysis revealed a hydrodynamic size of 50.79 nm and a surface charge of −17.49 mV, indicating good stability. Fourier transform infrared (FTIR) spectroscopy analysis identified functional groups (C=O, N-O, and O-H) that are crucial for nanoparticles stabilization. Scanning electron microscopy (SEM) and High-resolution transmission electron microscopy (HR-TEM) analysis revealed spherical, rod-shaped and hexagonal nanoparticles with sizes between 12 and 41 nm. Mycogenic-zinc oxide nanoparticles (M-ZnO NPs) significantly inhibited the mycelial growth of *A. brassicae* by 91.48% at 200 μg/mL, compared to chemically synthesized ZnO NPs at 200 μg/mL (79.62%) and mancozeb 0.2% (82.96%). SEM-EDX analysis revealed deformations and absorption of M-ZnO NPs in fungal hyphae, while confocal laser scanning microscopy (CLSM) showed increased reactive oxygen species (ROS) formation and impaired membrane integrity in treated fungal cells. Stress enzyme analysis confirmed increased superoxide dismutase (SOD) and catalase (CAT) activity by 44.2 U/mol and 39.6 U/mol at 200 μg/mL M-ZnO NPs. Our studies suggest that the M-ZnO NPs synthesized with *T. harzianum* culture filtrate have increased antifungal activity even at lower doses and can be used as an alternative to traditional fungicides without affecting environment.

## 1 Introduction

Nanotechnology holds great transformative potential in various areas such as drug delivery, cosmetics, industrial applications and agriculture (Zhang et al., 2008). This new technology takes advantage of distinct physical, chemical, and biological characteristics, such as increased surface area and reactivity, that materials exhibit at the nanoscale (1 to 100 nm). An important part in the domain of nanotechnology is the designing of nanoparticles which can be produced from different chemical, physical and biological approaches (El-Belely et al., 2021; Nehru et al., 2023; El-Moslamy et al., 2023). The physical and chemical approach for the synthesis of nanoparticles are not suitable as the process requires hazardous chemicals as well as extreme conditions i.e., pressure, energy and temperature and this procedure also produces hazardous by-products and nanoparticles having less stability (Shaheen et al., 2019; Aref and Salem, 2020; El-Belely et al., 2021; Nehru et al., 2023). Due to the limitations of physical and chemical approaches, the use of a biological approach or green nanotechnology is considered more reasonable because it uses an environmentally friendly approach, harmless chemicals and simple processes (El-Belely et al., 2021; Abdelkader et al., 2022; Nehru et al., 2023). The green synthesis of nanoparticles can be achieved by using various biological entities i.e., bacteria, fungi, cyanobacteria, actinomycetes, macro-algae, and plants (Salem and Fouda, 2021; El-Belely et al., 2021; Nehru et al., 2023).

Metal nanoparticles have attracted the most attention among all nanoparticle classes in agriculture because of their ability to interact with biological entities at the molecular level. Zinc oxides nanoparticles (ZnO NPs) have antimicrobial and antifungal properties, making them more effective nano pesticides than conventional pesticides (Malandrakis et al., 2018). ZnO nanoparticles are able to directly interact with the cell membranes of plant pathogens, resulting in structural damage. Its small size allows it to penetrate the cell walls of fungi and bacteria, leading to membrane depolarization, internal cell leakage, and ultimately cell lysis (Sirelkhatim et al., 2015). This occurred because zinc ions (Zn^2+^) are released into the environment as the ZnO NPs slowly dissolve. These zinc ions can disrupt important enzyme processes, protein synthesis and DNA replication, making them hazardous to microbial cells (Stoimenov et al., 2002). ZnO NPs have direct antifungal properties, however in plants they can also lead systemic resistance.

Mustard (*Brassica juncea*) is an important oilseed crop producing high-quality edible oil and protein-rich meal, enhancing global nutritional health and food security. Globally, it ranks third in terms of production after soybean and palm oil (FAO, 2021). However, foliar diseases significantly reduce mustard yield, and the extent of yield loss varies depending on disease severity, environmental factors, and management techniques. The most common foliar diseases that affect mustard include white rust (*Albugo candida*), powdery mildew (*Erysiphe cruciferarum*), downy mildew (*Peronospora parasitica*) and alternaria blight (*Alternaria brassicae*). Among all foliar diseases, alternaria blight is the most damaging foliar diseases affecting mustard. This disease can result yield losses ranging from 32 to 57% (Meena et al., 2010). Traditional methods, such as the application of fungicides and the use of resistant varieties, have been used to control Alternaria blight in mustard, these strategies often face difficulties in terms of long-term viability and environment sustainability (Abhilash and Singh, 2009). As a result, there is an increasing demand for more innovative and alternative approaches to combat fungal diseases. Nanotechnology offers new opportunities for sustainable disease management in agriculture. Lingaraju (2016) found that ZnO NPs had antifungal activity against *Alternaria alternata* in tomato plants by causing the destruction of fungal cell membranes and subsequent death of fungal cells. ZnO nanoparticles have shown potent antifungal activity against *Fusarium oxysporum* in tomatoes and cucumbers by inhibiting spore germination and mycelial growth (He et al., 2011).

The use of nanoparticles in agriculture also has some adverse effects, especially metal nanoparticles, which impose certain limitations on plants. High concentrations of metal and metal oxide nanoparticles, such as silver nanoparticles (AgNPs) and zinc oxide nanoparticles (ZnO), induce oxidative stress through the generation of reactive oxygen species (ROS), leading to lipid peroxidation, DNA damage and disruption of cellular structures (Rastogi et al., 2017). High concentrations of ZnO NPs (500 ppm) can cause phytotoxicity compared to lower concentrations, which adversely affects morphological traits such as reduction in root and shoot lengths in wheat seedlings (Pandya et al., 2024). Genotoxic effects, including chromosomal aberrations, micronuclei formation and reduced mitotic indices, have been observed in plants like *Vicia faba* exposed to AgNPs (Patlolla et al., 2012). In addition, the accumulation of nanoparticles in the environment increases the risk of exposure due to their potential to enter the food chain and pose a threat to higher trophic levels, including humans. Despite these challenges, nanoparticles at optimal concentrations can play a crucial role in controlling plant diseases by exhibiting antimicrobial properties that directly combat pathogens, thereby improving plant health.

The aim of the present study is to investigate the potential of *T. harzianum* in synthesizing zinc oxide nanoparticles (ZnO NPs) through a sustainable mycosynthesis approach. Fungi are extraordinary bio-factories because of their ability to secrete a wide range of biomolecules including enzymes, proteins and secondary metabolites, which act as reducing and stabilizing agents in nanoparticle synthesis. Zinc oxide nanoparticles are known for their unique physicochemical properties, including high surface area to volume ratio, catalytic efficiency and potent antimicrobial activities. However, concerns about their phytotoxic effects such as oxidative stress, disruption of photosynthesis processes and changes in nutrient uptake must be taken into account. The study also investigates the antifungal efficacy of M-ZnO NPs against *A. brassicae*. The antifungal activity of these nanoparticles was evaluated in a dose-dependent manner, as demonstrated by the significant reduction in fungal growth under *in vitro* conditions. Furthermore, M-ZnO NPs induced changes in fungal mycelium, spore structure, ROS generation and membrane integrity were also observed. To the best of our knowledge, Trichoderma-mediated synthesis of ZnO nanoparticles against *A. brassicae* is still unexplored and the influence of nanomaterials on the mechanism of toxicity to the pathogen remains to be discussed.

## 2 Materials and methods

The chemicals used in the experiments were of analytical grade and did not undergo further purification. Zinc acetate dihydrate [Zn (CH3.COO)2.2H2O] and chemically synthesized zinc oxide C-ZnO NPs (< 50 nm) of CAS no-1314-13-2 were obtained from Sisco Research Laboratories Pvt. Ltd., Maharashtra, India. The commercial fungicide Abic M-45 Mancozeb (75%) was used as standard control.

### 2.1 Isolation, purification and identification of fungal cultures

A soil sample was collected from the banana rhizosphere for the isolation of Trichoderma. Serial dilutions were performed on potato dextrose agar (PDA) media. Pure fungal colonies of Trichoderma were collected after 2 days and sub cultured on PDA at 28 ± 1°C. Alternaria was isolated from infected mustard leaves using PDA media. The pure culture of Alternaria was maintained at 28 ± 1°C and revived regularly as needed. The fungus was identified both morphologically and via a molecular approach. The ITS1 forward primer (5′-TCCTAGGTGAACCTGCGG-3′) and the ITS4 reverse primer (5′-TCCTCCGCTTATTGATATGC-3′) were used to amplify the ITS region. The DNA sequences were subjected to BLAST analysis through a BLAST search in GenBank (http://blast.ncbi.nlm.nih.gov/). The nucleotide sequences of *Trichoderma* sp. and *Alternaria* sp. was submitted to GenBank.

### 2.2 Filtrate-mediated synthesis of M-ZnO NPs

**Figure 1:**
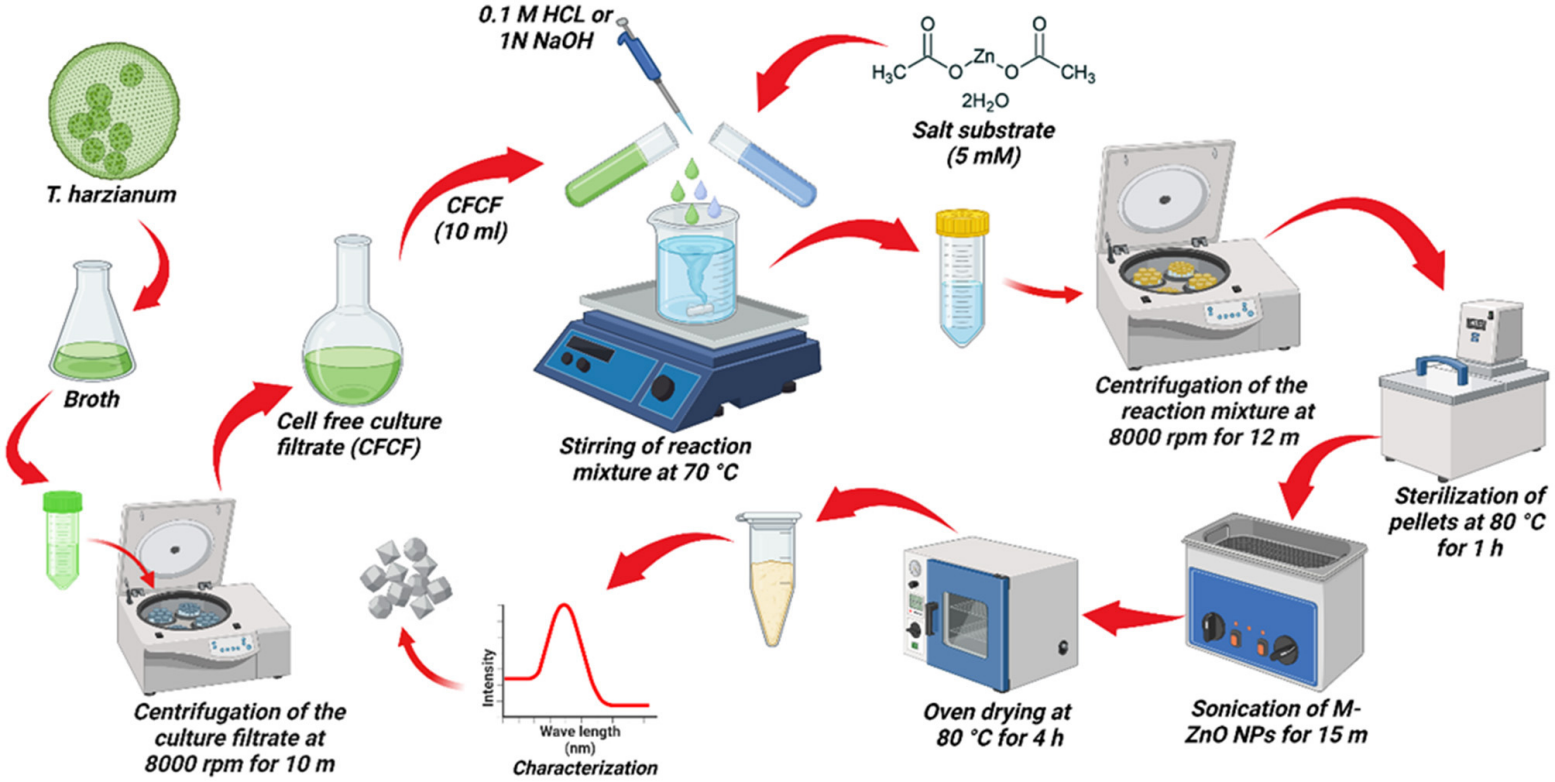
Pictorial representation of the myco-synthesis of ZnO NPs using *T. harzianum* culture filtrate.

*Trichoderma harzianum* was cultured on potato dextrose broth (PDB) medium at 28 ± 1°C for 7 days to produce fungal biomass. The *T. harzianum* biomass was then suspended in distilled water and stirred for 72 h at 140 rpm at 28°C. The cell-free culture filtrate (CFCF) from the fungal biomass was then separated by centrifugation at 8,000 rpm for 10 min. For the mycosynthesis of the ZnO NPs, a 50 mL substrate solution of zinc acetate dihydrate [Zn ^(CH3.COO)2.2H^2^O]^ at a concentration of 5 mM was prepared with distilled water. Afterwards, 10 ml of CFCF was added slowly to the substrate and pH of the reaction was maintained between 6 and 9 by adding either 0.1 M HCl or 1 N NaOH. The reaction mixture was stirred at different temperatures on a magnetic stirrer (Zaki et al., 2021). The CFCF and substrate solutions were kept in separate flasks as positive and negative controls, respectively. After 2 h, the color of the reaction mixture changed slightly, indicating the beginning of the nucleation phase. The reaction mixture was then kept on a rotary shaker at 140 rpm at 40°C for 12 h to completely reduce the substrate into nanoparticles. The UV spectra revealed an absorption band between 200 and 400 nm. A pictorial representation of this process is shown in Figure 1.

### 2.3 Optimization of physicochemical parameters for the mycosynthesis of ZnO nanoparticles

The nanoparticles were synthesized by optimizing various parameters such as CFCF concentration, metal salt concentration, pH and reaction temperature. The synthesis of nanoparticles is significantly influenced by these parameters. The effects of different concentrations of CFCF filtrate namely 5, 10, 15, and 20 ml on the mycosynthesis of nanoparticles were analyzed. The other parameters such as temperature, metal salt concentration and pH were held constant. The absorbance of the solution was recorded with a spectrophotometer. The reaction time for all parameters to be standardized was initially between 0 and 2 h. Later, the reaction mixture was shifted for 12 h at 40°C and 140 rpm for all parameters until the substrate was completely reduced and stabilized. To optimize the mycosynthesis protocol different concentrations of the metal salt (substrate) were used namely 1, 2, 4, and 5 mM, while the CFCF concentration, temperature and pH 9 were held constant. The optimization of the solution pH (6 to 9) was adjusted during the reaction process, while other variables such as the reaction temperature, CFCF concentration and substrate concentration was held constant. To optimize the reaction temperature, a temperature between 50 and 100°C was set during the reaction process, while the CFCF concentration, substrate concentration and pH 9 were held constant. The absorbance of each resulting solution for individual parameters was measured using spectrophotometer.

### 2.4 Purification of M-ZnO nanoparticles

To eliminate contamination and ensure maximum recovery of the M-ZnO nanoparticles, the obtained solution was centrifuged at 8,000 rpm for 12 min. The pellets were cleaned with 70% ethanol and rinsed three times with deionized water. The recovered pellets were redissolved in deionized water and sterilized in a water bath at 80°C for 1 h for three consecutive days. The M-ZnO NP solution was sonicated for 15 min to prevent aggregation of the nanoparticles. Finally, the solution was dried at 80°C for 4 h and then stored at room temperature for further use.

### 2.5 Characterization of nanoparticles

#### 2.5.1 Ultraviolet-visible spectroscopy

The synthesized M-ZnO nanoparticles were first characterized using a UV-Vis spectrophotometer (Hitachi, U-3900, version 2J2530004, Japan). Since it measures the optical properties of nanoparticles. The absorption spectra of M-ZnO NPs were observed in the range 200–800 nm. Distilled water was kept as a blank.

#### 2.5.2 X-ray diffraction analysis

For the crystal structure and size of ZnO nanoparticles, powdered samples were subjected to XRD (Rigaku Ultima IV) with kα radiation (λ = 1.5406 Å), an operating voltage of 30 kV and a scanning speed of 3° per minute, covering the 2θ range from 10 to 60°C. The average particle size was calculated using the formula proposed by Scherrer (Thein et al., 2017; Mustapha et al., 2019).


$$\begin{array}{l} \text{D }=\text{ k}\lambda /\beta \cos\theta \end{array}$$

where D stands for the average crystal size, K for the Scherrer constant (K = 0.94), λ for the wavelength of the X-ray (1.5406 Å) and β for the full width at half maximum (FWHM) the diffraction peak (determined with Origin Pro, 2024) and θ is the Bragg angle. The d distance value was determined using the Bragg equation (Jamdagni et al., 2018).


$$\begin{array}{l} \text{d }=\text{ n}\lambda /2\sin\theta \end{array}$$

where *d* is the distance between diffraction planes, *n* is the order of reflection, λ is the wavelength of the X-ray (1.5406 Å), and θ is the angle of incidence. Subsequently, Panalytical X'Pert High Score software was used to determine crystal structure.

#### 2.5.3 Dynamic light scattering and zeta potential analysis

The surface charge of M-ZnO NPs, hydrodynamic size, polydispersity index, and particle size distribution were determined using DLS and zeta potential (Zetasizer, Nano ZS). The dried powder of M-ZnO NPs was suspended in deionized water and subjected to probe sonication for 30 min. Later, the sample was carefully transferred to a cuvette and placed in the device, keeping the temperature equilibration at 25°C for 1 min. Data were collected and analyzed.

#### 2.5.4 Scanning electron microscopy with EDX analysis

The surface morphology, size and shape of the M-ZnO NPs were determined by SEM. The presence of zinc elements in the nanoparticles and their chemical composition were confirmed using EDX analysis. The powdered sample of M-ZnO NPs was placed on the carbon-coated grid and spread evenly. Ultra-thin gold coating was performed on the carbon tape along with the nanoparticles using a sputter coating machine and observation were taken under SEM (Zeiss EVO40).

#### 2.5.5 High-resolution transmission electron microscopy

To confirm the size and particle size distribution of M-ZnO NPs, HR-TEM (JEOL 2100F) was used. Samples of nanoparticles sonicated for 30 min were placed on a carbon-coated copper grid with a mesh size of 400 and then stained with a 2% solution of uranyl acetate. The sample was allowed to air dry for 1 h at room temperature and then visualized in TEM. The average particle size was then estimated using the “image J and Origin Pro, 2024” software.

#### 2.5.6 Fourier transform infrared spectroscopy and thermogravimetric analysis

The surface functional groups and chemical composition of the M-ZnO NPs were evaluated using FTIR spectroscopy (Bruker, Tensor 37, Germany). FTIR analysis was performed on a translucent KBr pellet obtained by pressing the sample at 6,000 kg cm^−1^ under high pressure for 2 min after adding 100 mg of spectral grade KBr. Changes in the thermal stability of the nanoparticles were measured using TGA. A furnace was filled with M-ZnO NPs, which were then heated to a specific temperature. The temperature-dependent weight loss percent were used to estimate the data.

### 2.6 Effect of M-ZnO NPs on *A. brassicae* under *in-vitro* conditions

The antifungal properties of M-ZnO NPs were evaluated against *A. brassicae* by using poisoned food techniques. The stock solutions of M-ZnO and C-ZnO NPs were added to 20 milliliters of PDA at different concentrations. Different concentrations of 10, 25, 50, 100, 150, and 200 μg of M-ZnO or C-ZnO NPs per ml of PDA were prepared. A PDA plate with the commercial fungicide mancozeb (0.2%) served as a positive control, whereas a PDA plate without M-ZnO NPs served as negative control. A 4 mm fungal disc was cut from a 7-day-old culture of *A. brassicae* and placed in the center of each PDA plate. The Petri dishes were subsequently sealed and kept in an incubator at 28 ± 1°C until growth in the negative control plates reached the periphery. Three replicates were performed for each treatment. After 10 days, radial growth was measured and a photograph was taken. The following formula was used to calculate the percentage of radial fungal growth inhibition for each treatment compared to the control.


$$\begin{array}{l} \text{Percent Inhibition }\left(\%\right)\;=\;\left[\left(\text{C}-\text{T}\right)\right]/\left[\text{C}\right]\;\times \;100 \end{array}$$

where, C = control, T = treatment.

### 2.7 Effect of M-ZnO NPs treatment on conidia

To observe morphological changes in fungal conidia, *A. brassicae* was grown on PDA plates containing different concentrations, namely 10, 25, 50, 100, 150, 200 μg/mL M-ZnO NPs and fungicides (0.2%). The Petri dishes were then incubated at 28 ± 1°C for 7 days. Conidia were examined under a light microscope (Carl Zeiss, Germany) at 40 × magnification for length and width as well as for the percentage of conidial damage, and the results were compared between the treated and control plates. This study was conducted in triplicate. The following formula was used to calculate the percentage of spore damage:


$$\begin{array}{l} \text{Spore damage percentage }=\;\frac{\text{Damaged spores }\times \;100}{\text{Total no}.\text{ of spores}} \end{array}$$

### 2.8 Surface stability of M-ZnO nanoparticles on fungal hyphae through zeta potential determination

To analyze the interaction between *A. brassicae* hyphae and M-ZnO nanoparticles, equal volumes of spore suspensions were cultured overnight in PDB media supplemented with different concentrations (10, 25, 50, 100, 150 and 200 μg/mL) of M-ZnO NPs at 28 ± 1°C. The zeta potentials of both the fungal cell suspension alone and the combination of the fungal cell suspension with M-ZnO NPs at different concentrations were subsequently analyzed using a zeta potential analyzer (Zetasizer, Nano ZS).

### 2.9 Assessing morphological changes in fungal mycelia through SEM and EDX

SEM was employed to examine the morphological alterations in fungal hyphae induced by M-ZnO NPs in fungal hyphae. Briefly, the mycelia of *A. brassicae* were collected from M-ZnO NPs treated and untreated PDA plates. The collected mycelium was fixed in glutaraldehyde (2.5%) at 4°C for 4 h and then rinsed with 0.5 M phosphate buffer (PBS) solution (pH = 7). Using a gradient series of ethanol (30: 50: 70: 80: 90: 100%), the samples were dehydrated for 5 min at each gradient. The dehydrated samples were finally oven-dried at 40°C, the mycelia were placed on carbon-coated stubs and gold coating was performed using a sputter coater. SEM (Zeiss EVO 40) was used to observe any changes in morphology.

### 2.10 Estimation of ROS in fungal hyphae treated with M-ZnO NPs

The oxidative stress in fungal hyphae exposed to M-ZnO NPs was detected using the fluorescent probe 2′,7′-dichlorodihydrofluorescein diacetate (H2DCFDA) following the method described by Egan et al. (2007) with some modifications. The mycelia of *A. brassicae* were cultured on PDB supplemented with 100, 150 and 200 μg/mL M-ZnO NPs for 24 h at room temperature (RT). The hyphae were washed by centrifugation with PBS at 4,000 rpm for 5 min. Afterwards, the sample was stained with 20 μM H2DCFDA for 30 min in the dark at room temperature. The production of ROS in the fungal hyphae was observed using confocal laser scanning microscopy (CLSM, Leica), with excitation and emission wavelength of 490 and 525 nm, respectively. The percent ROS fluorescence intensity in the M-ZnO NPs- treated and untreated hyphal images was quantified using ImageJ software (Rossi et al., 2017).

### 2.11 Determination of plasma membrane integrity in fungal hyphae treated with M-ZnO NPs

The plasma membrane integrity of *A. brassicae* treated with M-ZnO NPs was observed using propidium iodide (PI; membrane-impermeable dye) following the protocol of Setiawati et al. (2017) with some modifications. The mycelium of the fungus was collected and prepared following the protocol described above. An untreated mycelium served as a control. Subsequently, 10 μg/ml PI was used to stain the collected mycelium for 20 min at RT in the dark. The mycelium was then rinsed with 0.5 M PBS and resuspended in the same solution. The membrane damage caused by the M-ZnO NPs in the fungal hyphae was visualized under CLSM with emission and excitation at 617 and 536 nm, respectively. The fluorescence intensity of damaged membrane was quantified using ImageJ software.

### 2.12 Assessment of stress related enzymes in *A. brassicae* treated with M-ZnO NPs

The superoxide dismutase activity of fungal mycelia exposed to M-ZnO NPs was analyzed according to the method proposed by Beauchamp and Fridovich (1971). The treated mycelium was harvested, rinsed with distilled water, and then crushed with PBS (pH 7.0). The homogenized mixture was centrifuged at 15,000 × g for 10 min at 4°C to obtain the enzyme extract. The 3 ml assay mixture was prepared (13 mM methionine, 2 mM riboflavin, 0.1 mM EDTA, 75 μM NBT, 50 mM PBS, pH 7.8) with the enzyme extract. These samples were incubated under a fluorescent lamp for 30 min. The absorbance was measured at 560 nm as described by Raghib et al. (2020). The activity of catalase in fungal mycelia treated with M-ZnO NPs was examined using the Aebi (1984) method. Briefly, 0.5 mL of enzyme extract was added to 3 mL of reaction mixture containing, 20 mM H_2_O_2_ and 50 mM phosphate buffer (pH-7) and absorbance was measured at 240 nm (Raghib et al., 2020).

### 2.13 Statistical analysis

The experiments were performed following complete randomized design (CRD) with three repetitions. The statistical data generated in the experiment were analyzed using R software version 4.4.1. (Foundation for Statistical Computing, Vienna, Austria). After analysis of variance (ANOVA), Tukey's multiple comparison *post-hoc* test (p ≤ 0.05) was used to identify significant differences between treatment means. The statistical graphs were created using GraphPad Prism 8.0.1 and OriginPro 2024.

## 3 Results

### 3.1 Isolation, purification and identification of fungi

Soil was collected from the banana rhizosphere in Jobner district, Rajasthan for isolation and purification of *Trichoderma spp*. on PDA media (Zhou et al., 2020). The *Alternaria spp*. infected mustard leaves were collected from Prayagraj district, Uttar Pradesh, India and the pathogenic fungi were isolated and purified on potato dextrose medium (PDA) according to previously described method (Meena et al., 2017). Cultural, morphological and microscopic observations were used to identify both fungi, and similar work was performed by Sharma et al. (2013). Both fungi were identified as *Trichoderma harzianum* and *Alternaria brassicae* on the basis of their morphological and cultural characteristics. The fungi isolated from banana rhizosphere and mustard diseased leaves were subjected to molecular identification, and confirmed by a BLAST search at the NCBI (Bethesda, MD, USA). On this basis, the species were assigned to them as *Trichoderma harzianum* and *Alternaria brassicae* with 94.81% and 99.26% identity, respectively. The following accession numbers PP345607 and PP345617 were obtained for *T. harzianum* and *A. brassicae*, respectively. Similar molecular identification work was performed by Tomah et al. (2020) for *Trichoderma* spp. and for identifying *A. brassicae* (Sharma et al., 2013).

### 3.2 Synthesis of M-ZnO NPs

The synthesis of metal nanoparticles using *Trichoderma* spp. is a green chemistry approach in which fungal metabolites act as reducing and stabilizing agents (Zaki et al., 2021). This biosynthesis method is environmentally friendly as it does not require toxic chemicals. Trichoderma produces metabolites and enzymes that reduce Zn^2+^ ions to ZnO nanoparticles (Consolo et al., 2020). The reaction mechanism can be simplified as follows:


(1) $$\begin{array}{l} \text{Zn }{\left(\text{CH}3\text{COO}\right)}_{2}.\;2\text{H}2\text{O }\to \text{ Z}{\text{n}}^{2+}\;+\;2\text{CH}3\text{COO }+\;2{\text{H}}_{2}\text{O} \end{array}$$

(2) $$\begin{array}{l} \text{Z}{\text{n}}^{2+}\;+\text{ Fungal metabolites }\to \text{ ZnO NPs }+\text{ Byproducts} \end{array}$$

### 3.3 Physicochemical parameters of the M-ZnO nanoparticles

The synthesized nanoparticles presented an absorption peak at approximately 200–400 nm. The obtained absorption peaks of the synthesized nanoparticles are in accordance with those of earlier studies (Sumanth et al., 2020; Zaki et al., 2021), where the absorption peak was observed between 200 and 800 nm. Fungal-mediated nanoparticle synthesis has several benefits, such as the ability to form stable caps from fungal biomolecules that can support a range of biological activities. For the synthesis of ZnO nanoparticles, the culture filtrate of *Trichoderma harzianum* was employed as a reducing and stabilizing agent by adjusting different physicochemical parameters. For the mycosynthesis of ZnO nanoparticles, various *Trichoderma* spp. have been used. Previously, *T. asperellum* was used as a potential candidate for the synthesis of ZnO NPs (Shobha et al., 2023).

#### 3.3.1 Effect of culture filtrate concentration

**Figure 2:**
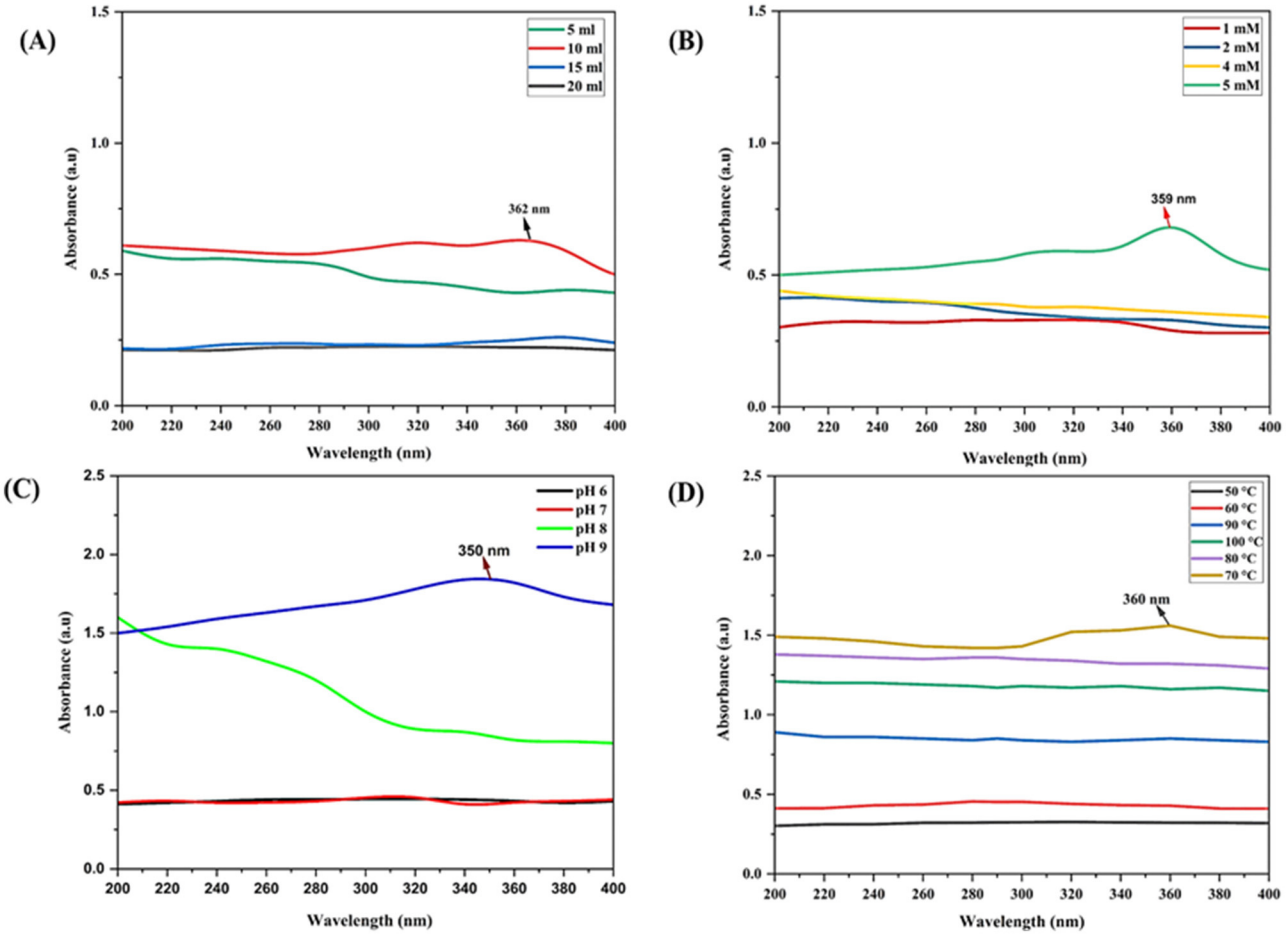
UV-Vis spectrum for the optimization of physicochemical parameters for myco-synthesis of ZnO NPs: **(A)** effect of CFCF concentration on synthesis of M-ZnO NPs, **(B)** effect of substrate concentration on synthesis of M-ZnO NPs, **(C)** effect of pH on the synthesis of M-ZnO NPs, **(D)** effects of temperature on the synthesis of M-ZnO NPs.

A variety of factors significantly contribute to the synthesis of nanoparticles. The effects of culture filtrates with different concentrations *viz.*, 5, 10, 15 and 20 ml were evaluated. The slightly low concentration of Trichoderma culture filtrate with zinc salt reacted to form nanoparticles with relatively high absorbance at 362 nm (Figure 2A). The solution began to form aggregates when the culture filtrate concentration was decreased or increased from 10 ml. This led to the conclusion that 10 ml of culture filtrate represented the optimal absorbance. Higher concentrations change the physiochemical properties of ZnO nanoparticles.

#### 3.3.2 Effect of substrate concentration

Different concentration of the substrate Zn (CH3.COO)2.2H_2_O (1, 2, 4 and 5 mM) were used to obtain optimal absorption peaks. Figure 2B shows that no absorption peak was present between 1 and 4 mM. Furthermore, as the concentration increased slowly, the characteristic absorption peak at 359 nm was at a concentration of 5 mM. Thus, the concentration of the substrate influences the nucleation of nanoparticles.

#### 3.3.3 Effect of pH

Few studies have investigated how pH affects ZnO nanoparticle synthesis. Here, different pH values were investigated for the synthesis of ZnO nanoparticles. Figure 2C shows that no absorption peak was observed at lower pH values between 6 and 9. At low pH, the aggregation of zinc nanoparticles results in larger nanoparticles rather than nucleation. The absorption peak at 350 nm at pH 9 meant that the substrate was completely reduced to zinc nanoparticles.

#### 3.3.4 Effect of temperature

To determine the effects of temperature, a solution containing 5 mM Zn (CH3.COO)2.2H2O) and 10 ml of *T. harzianum* culture filtrate was prepared. The results revealed that no absorption peak was found in the temperature range below 70°C and above 70°C, the absorption peak was critically found at 70°C at 360 nm (Figure 2D). Therefore, it is speculated that the bulk zinc acetate dihydrate was converted into zinc nanoparticles at 70°C.

### 3.4 Characterization of synthesized nanoparticles

#### 3.4.1 XRD analysis of the M-ZnO NPs

**Figure 3:**
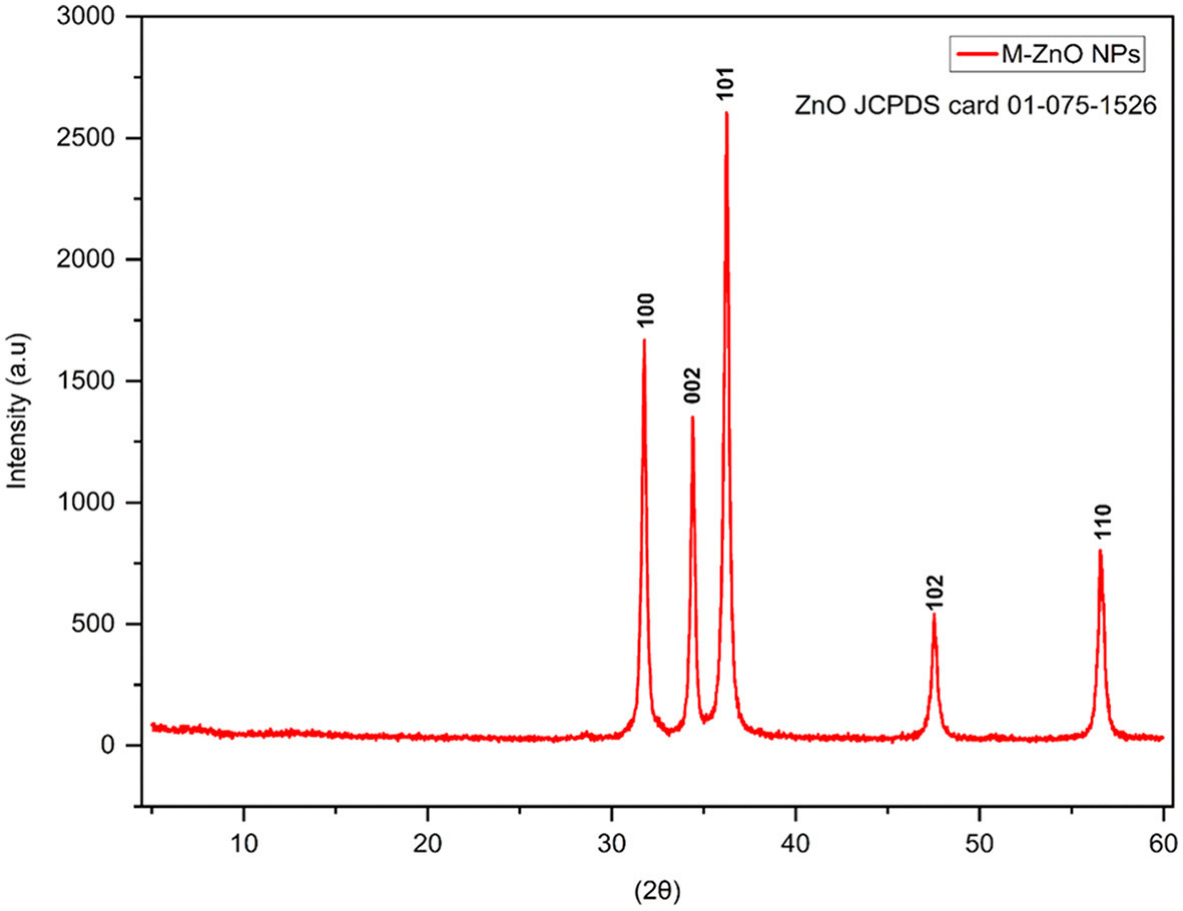
X-ray diffraction pattern of M-ZnO NPs.

XRD patterns of the synthesized ZnO nanoparticles recorded at 2θ angles ranging from 20 to 60°. The diffraction peaks at 2θ angles of 31.78, 34.4, 36.24, 47.54, and 56.56° corresponded to Miller indices (*hkl*) (100), (002), (101), (102) and (110), respectively (Figure 3, Supplementary Table 1). The JCPDS card number. 01-075-1526 was used to index the peaks of the M-ZnO NPs. The crystal structure of the synthesized nanoparticles was then determined using Panalytical X'Pert High Score software, which revealed the hexagonal structure of the M-ZnO NPs. The *d*-spacing of the synthesized M-ZnO NPs were 2.813, 2.601, 2.475, 1.912 and 1.625 Å. The crystallite particles were found to have an average size of 29.448 nm.

#### 3.4.2 DLS and zeta potential analysis

**Figure 4:**
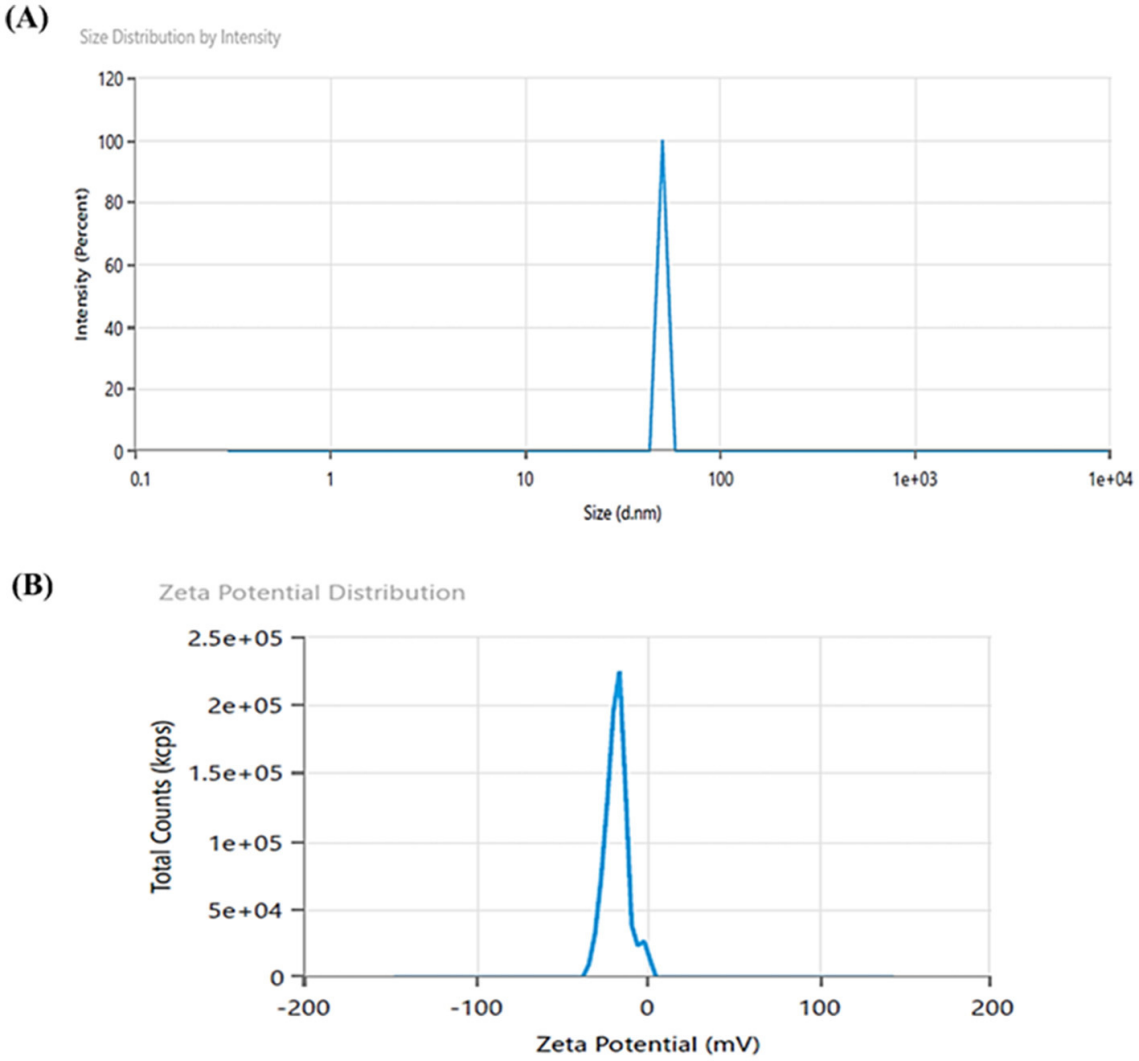
DLS image showing hydrodynamic size **(A)**, and zeta potential **(B)** of M-ZnO NPs.

DLS analysis confirmed the formation of zinc oxide nanoparticles with an average particle size of 50.79 nm and a polydispersity index of 1 (Figure 4A). M-ZnO NPs were found to be polydisperse in nature. Particles with a narrow size distribution indicate a polydispersity index ≤ 0.3. These results also confirm the polydisperse nature of synthesized nanoparticles as suggested by Lizunova et al. (2017) and Aldalbahi et al. (2020). DLS is known to provide a significantly larger particle size than TEM analysis because of the additional hydrate layers on the nanoparticle surface (Sattari et al., 2020). To further validate the actual size and morphology of the synthesized nanoparticles, TEM analysis was performed. Zeta potential analysis revealed that the surface charge of the synthesized nanoparticles was −17.49 mV (Figure 4B). This result shows that the majority of capping molecules present on the surface of synthesized nanoparticles are negatively charged groups.

#### 3.4.3 FTIR analysis of M-ZnO NPs

**Figure 5:**
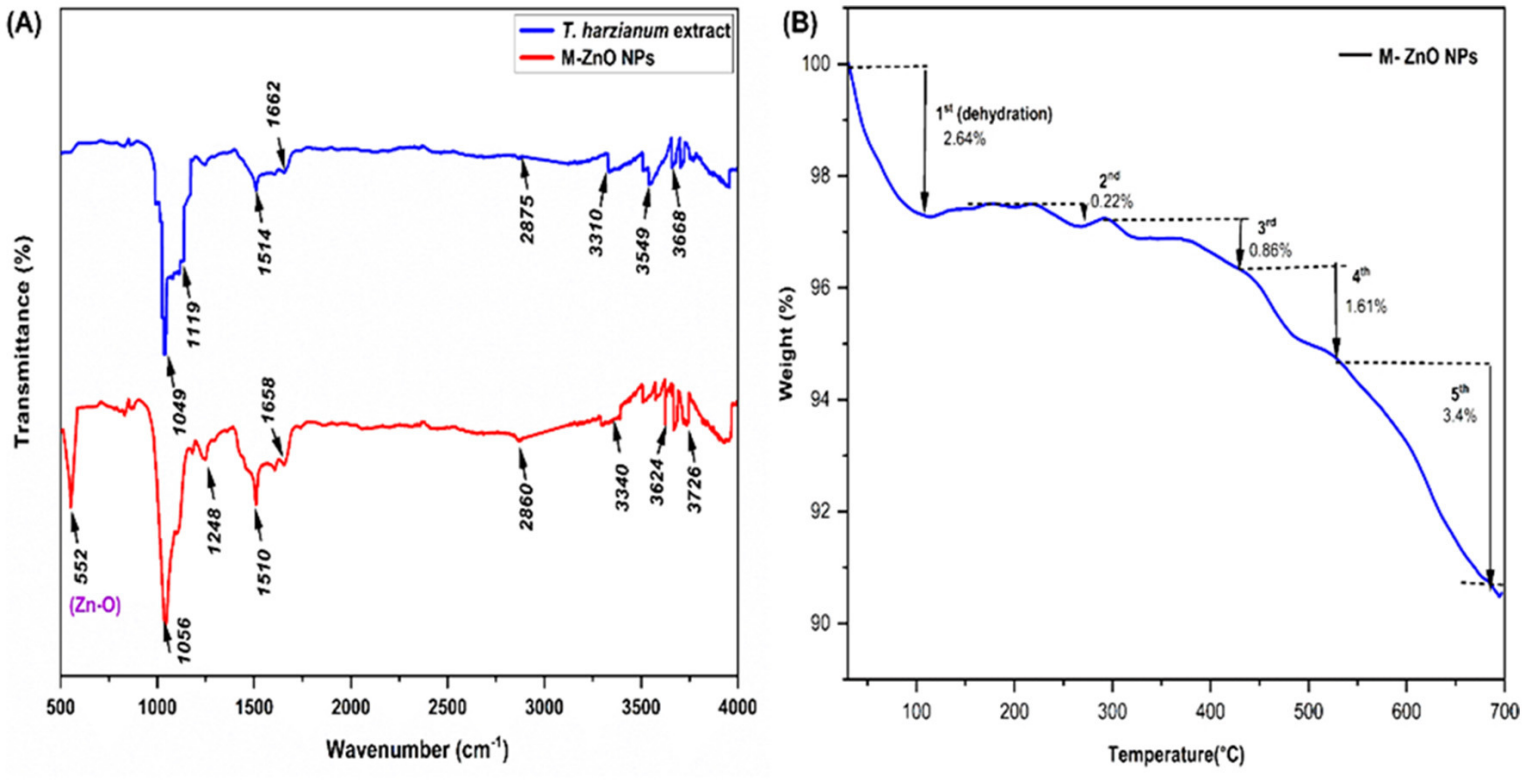
FTIR spectra for myco-synthesized ZnO nanoparticles **(A)**, TGA analysis **(B)** of M-ZnO NPs.

The FTIR spectra of the M-ZnO NPs synthesized from *T. harzianum* extract and dried are shown in Figure 5A, Supplementary Table 2. The peaks of the synthesized ZnO nanoparticles from *T. harzianum* were found at 552, 1,056, 1,248, 1,510, 1,658, 2,860, 3,340, 3,624, and 3,726 cm^−1^ in the FTIR spectrum, whereas the peaks of the *T. harzianum* extract were observed at 1,049, 1,119, 1,514, 1,662, 2,875, 3,310, 3,549 and 3,668 cm^−1^. The observed peak at around 552 cm^−1^ was recognized as a characteristic Zn-O band, which is consistent with findings from previous studies (Thongam et al., 2019). The absorption peaks corresponding to 1,056 and 1,248 M-ZnO NPs and 1119 *T. harzianum* can be attributed to C=O (carboxylic acid). Trichoderma produces a variety of secondary metabolites or organic acids that possess C=O and have antifungal properties. C=O is essential for enzyme-substrate binding and interactions. The C=O group help stabilize nanoparticles by acting as a capping agent. The peak at position 1,049 of *T. harzianum* appeared due to CO-O-CO stretching (anhydride). The stabilizing effect of the ester group on nanoparticles helps maintain their dispersion in solution by preventing agglomeration. The peaks of the M-ZnO NPs and *T. harzianum* that appeared at 1,510 and 1,514 were attributed to N-O (nitro compound) bands. The presence of N-O facilitates the reduction reaction by contributing electrons, which aids in the conversion of metal salts into nanoparticles. The peak at 1,658 cm^−1^ in M-ZnO NPs and 1,662 cm^−1^ in *T. harzianum* confirms the presence of a C=C band (alkene), as phenolic groups are found in the Trichoderma extract. The capping effect on the surface of M-ZnO NPs was achieved by phenolic compounds in the culture filtrate. The appearance of peaks at 2,860 (amine salt), 3,340 (aliphatic primary amine) and 2,875 (amine salt) for the M-ZnO NPs and *T. harzianum* was due to the N-H stretching band. However, the peak of *T. harzianum* at 3,310 cm^−1^ was due to N-H (secondary amine). The intense peaks at 3,624 and 3,726 cm^−1^ for the synthesized NPs and at 3,549 and 3,668 cm^−1^ for *T. harzianum* can be assigned to the O-H stretching bands. This O-H group facilitates the conversion of metal salts into zero-valent metal nanoparticles. The FTIR spectra revealed that alkaloid, flavonoid and phenol compounds were abundant in the *T. harzianum* extract and M-ZnO NPs.

#### 3.4.4 Thermogravimetric analysis (TGA)

TGA analysis of the synthesized M-ZnO nanoparticles revealed that weight loss continued until the temperature reached 696°C (Figure 5B). Initially, a weight loss 2.64% was observed between 30 and 108°C, which was most likely due to the evaporation of the moisture adsorbed on the surface of the M-ZnO NPs (Moharram et al., 2014). A gradual weight loss of 0.22% occurred between 108 and 272°C, which was attributed to the presence of adsorbed oxygen species. In the temperature range of 272 to 427°C, additional degradation occurred, resulting in a weight loss of about 0.86%, which was attributed to the desorption of organic compounds on the surface of the nanoparticles (Tilahun et al., 2023). A significant weight loss of 1.61% was recorded between 272 and 530°C, which was likely due to the degradation of phenols and flavonoids derived from the Trichoderma extract which contributed to the stabilization of the M-ZnO nanoparticles (Faisal et al., 2021). Above 530°C, a constant weight loss of approximately 3.4% was observed, which may be due to the thermal degradation of resistant aromatic compounds on the surface of the M-ZnO NPs (Khan et al., 2023). Ultimately, the residual weight of the M-ZnO nanoparticles at 696°C was 91.07% of the original weight.

#### 3.4.5 SEM and EDX analysis of M-ZnO NPs

**Figure 6:**
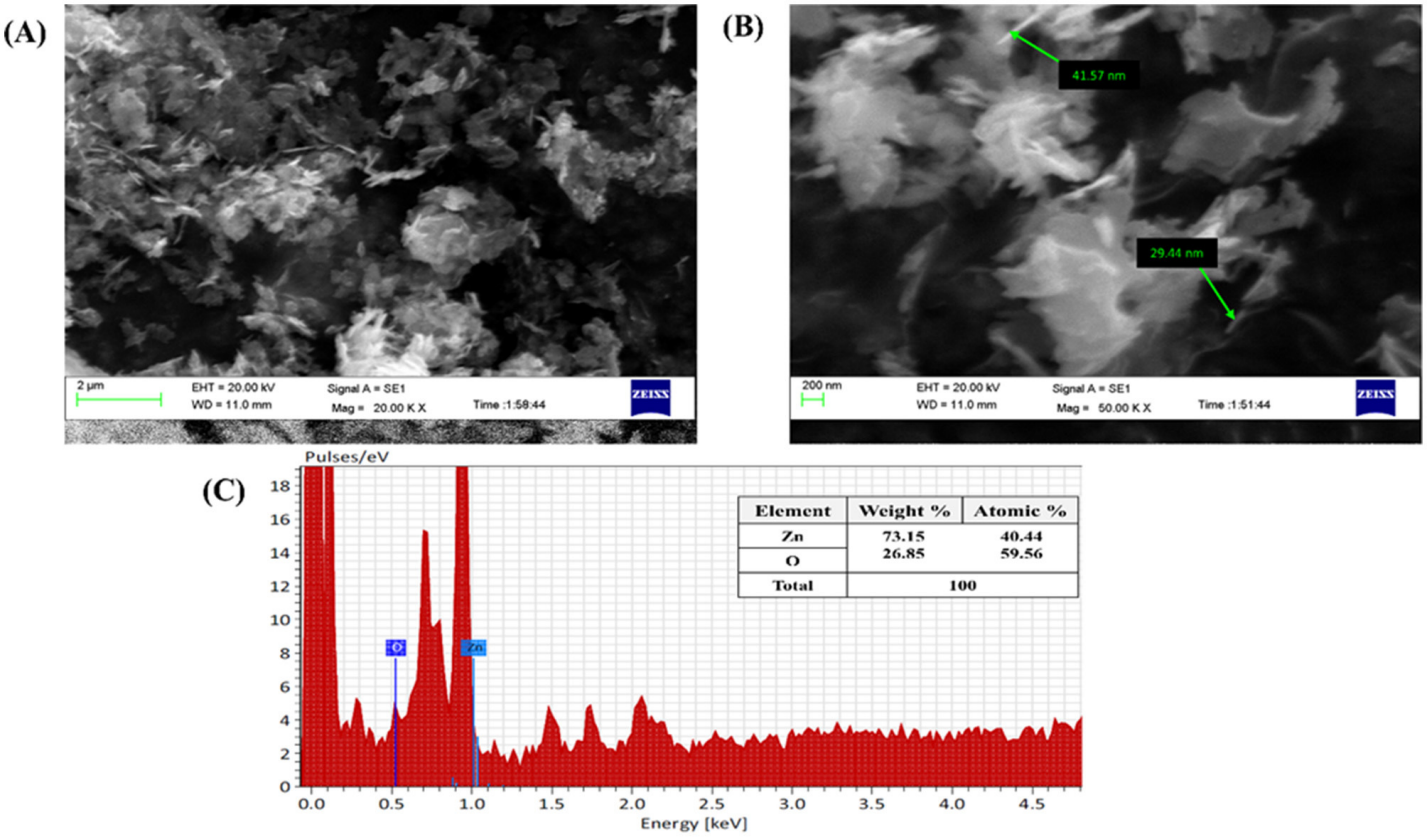
Microscopic analysis of M-ZnO NPs: **(A, B)** SEM images of M-ZnO NPs, and **(C)** EDX analysis of M-ZnO NPs.

The synthesized nanoparticles were subjected to SEM analysis which confirmed the presence of spherical, rod, and hexagonal shapes, as well as well-dispersed M-ZnO nanoparticles with some aggregation (Figures 6A, B) and microscopic images of the M-ZnO nanoparticles at different magnifications are shown. A study of the nanomaterials revealed that the particle sizes varied between 29 and 41 nm. EDX analysis showed that the weight percentages of oxygen and zinc were 26.85 and 73.15%, respectively. The atomic percentages of oxygen and zinc were 59.56 and 40.44%, respectively (Figure 6C). The absorption band was observed within the range of 1–5 keV.

#### 3.4.6 HR-TEM analysis of M-ZnO NPs

**Figure 7:**
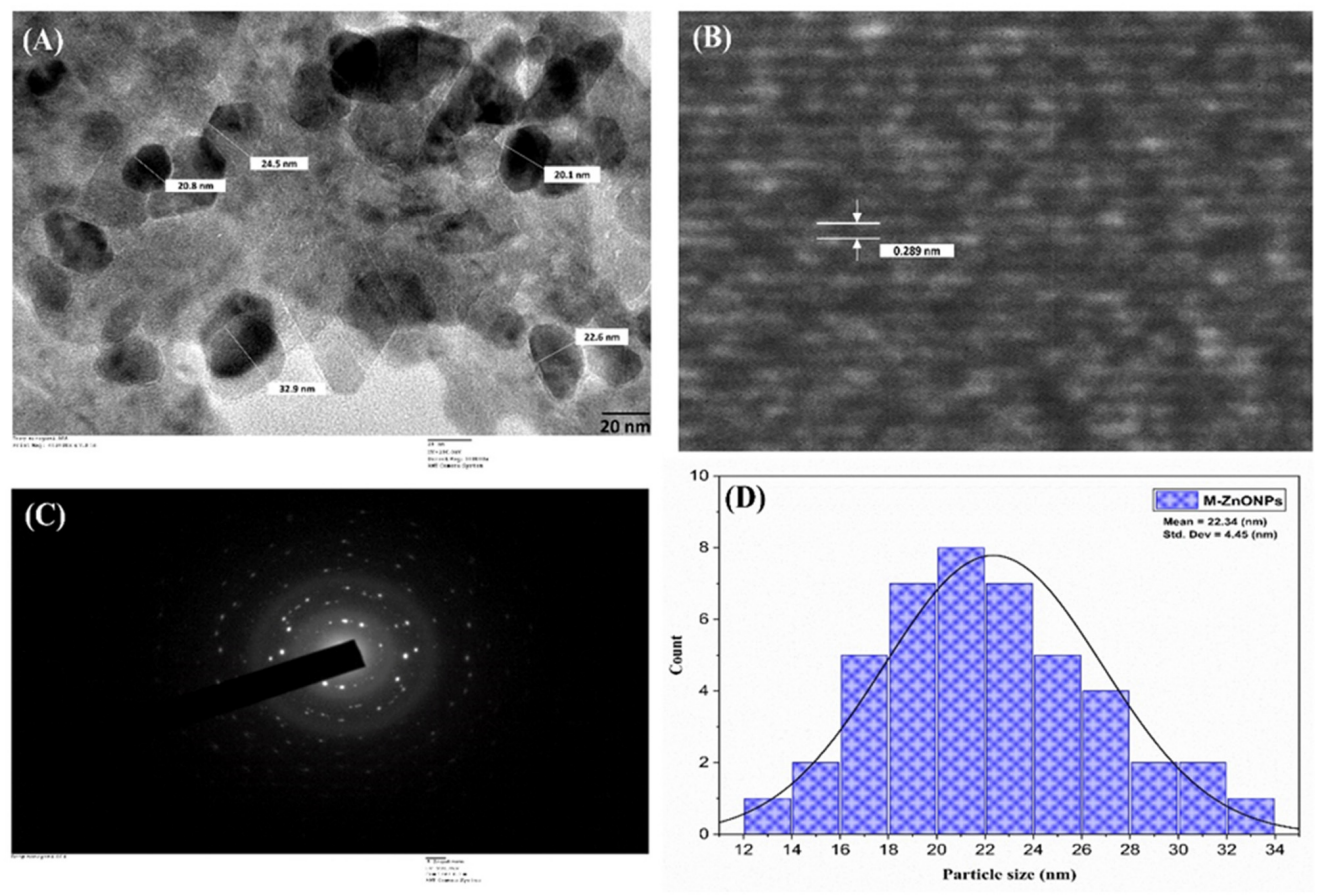
HR-TEM image of M-ZnO NPs **(A)**, lattice fringe of M-ZnO NPs **(B)** selected-area electron diffraction (SAED) pattern of M-ZnO NPs **(C)**, and **(D)** histogram of particle size distribution.

The internal structure of the M-ZnO nanoparticles was examined using TEM, and the images are shown in Figure 7A. The TEM images of the M-ZnO NPs show that many of the particles have a hexagonal shape with slight variations, which is consistent with the SEM findings. HR-TEM analysis clearly revealed the lattice fringes of the synthesized M-ZnO nanoparticles, which had a d-spacing of 0.289 nm (Figure 7B) and corresponded to the (100) plane (JCPDS card No. 01-075-1526). The SAED pattern (Figure 7C) shows reflections corresponding to the (100), (002), (101), (102) and (110) planes, which indicate an HCP structure and are consistent with the XRD results. Histogram analysis revealed that the average particle size was between 12 and 34 nm (Figure 7D). The particle size obtained from the HR-TEM analysis is largely consistent with that determined by XRD. The HR-TEM and XRD results are in complete agreement.

### 3.5 Antifungal effects of M-ZnO nanoparticles

The development of resistance among fungal pathogens to traditional fungicides has prompted the search for more effective and environmentally benign antifungal agents. As a result, nanoparticles (NPs) have gained attention in agriculture as promising “innovative-generation fungicides” (Ramezani et al., 2019).

**Figure 8:**
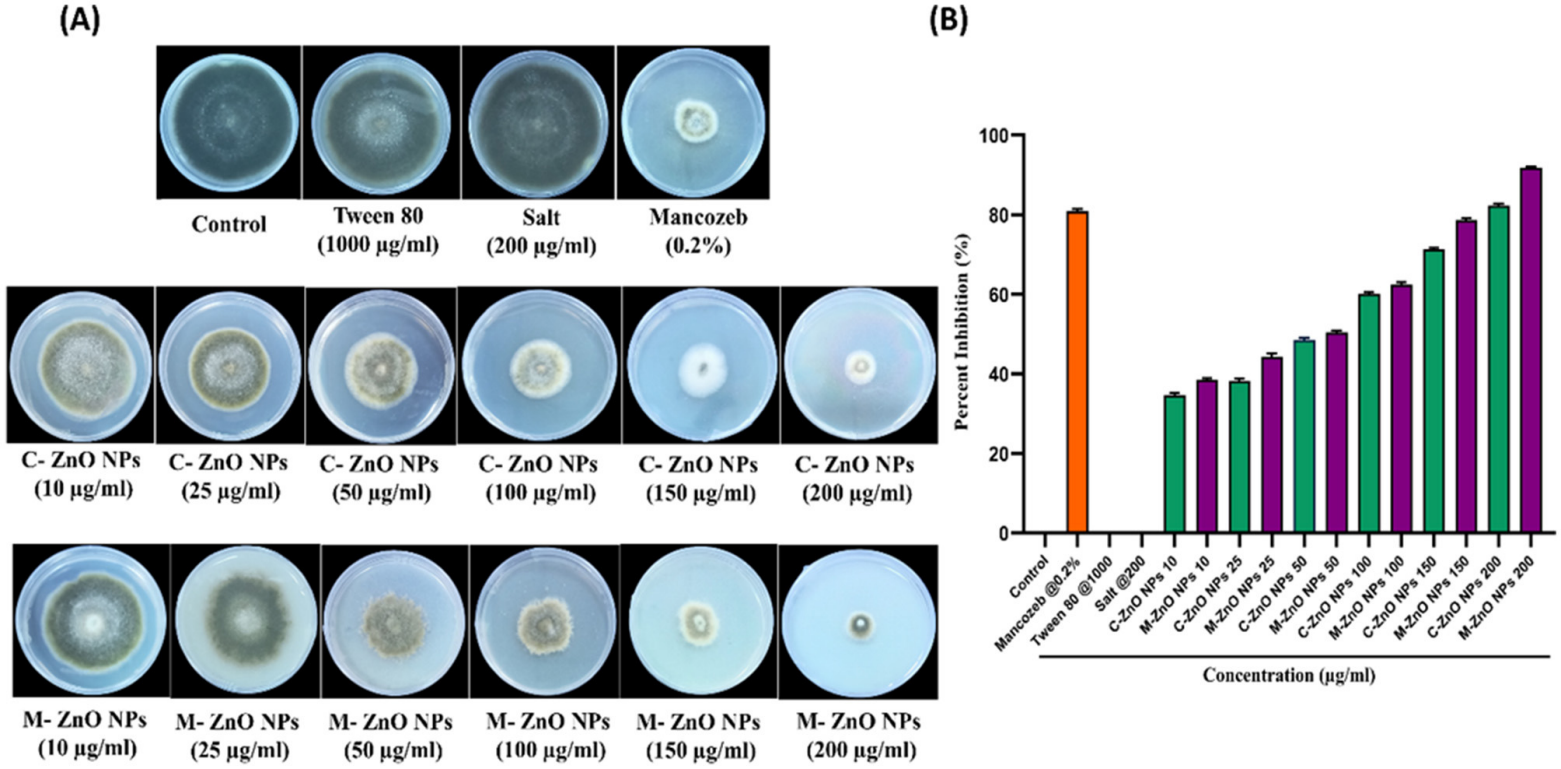
Effect of M-ZnO NPs and C-ZnO NPs on inhibition of radial mycelial growth of *A. brassicae* at different concentrations (10–200 μg/ml) in parallel with control, Tween 80, salt and fungicide treatment after 12 days post treatment. **(A)** Antifungal activity of M-ZnO NPs and C-ZnO NPs in PDA plates, **(B)** percentage inhibition graph of M-ZnO NPs and C-ZnO NPs on mycelial growth of A. brassicae. Data represents mean ± standard errors of three replicates by one-way-ANOVA (Tukey, HSD, *p* ≤ 0.05).

**Figure 9:**
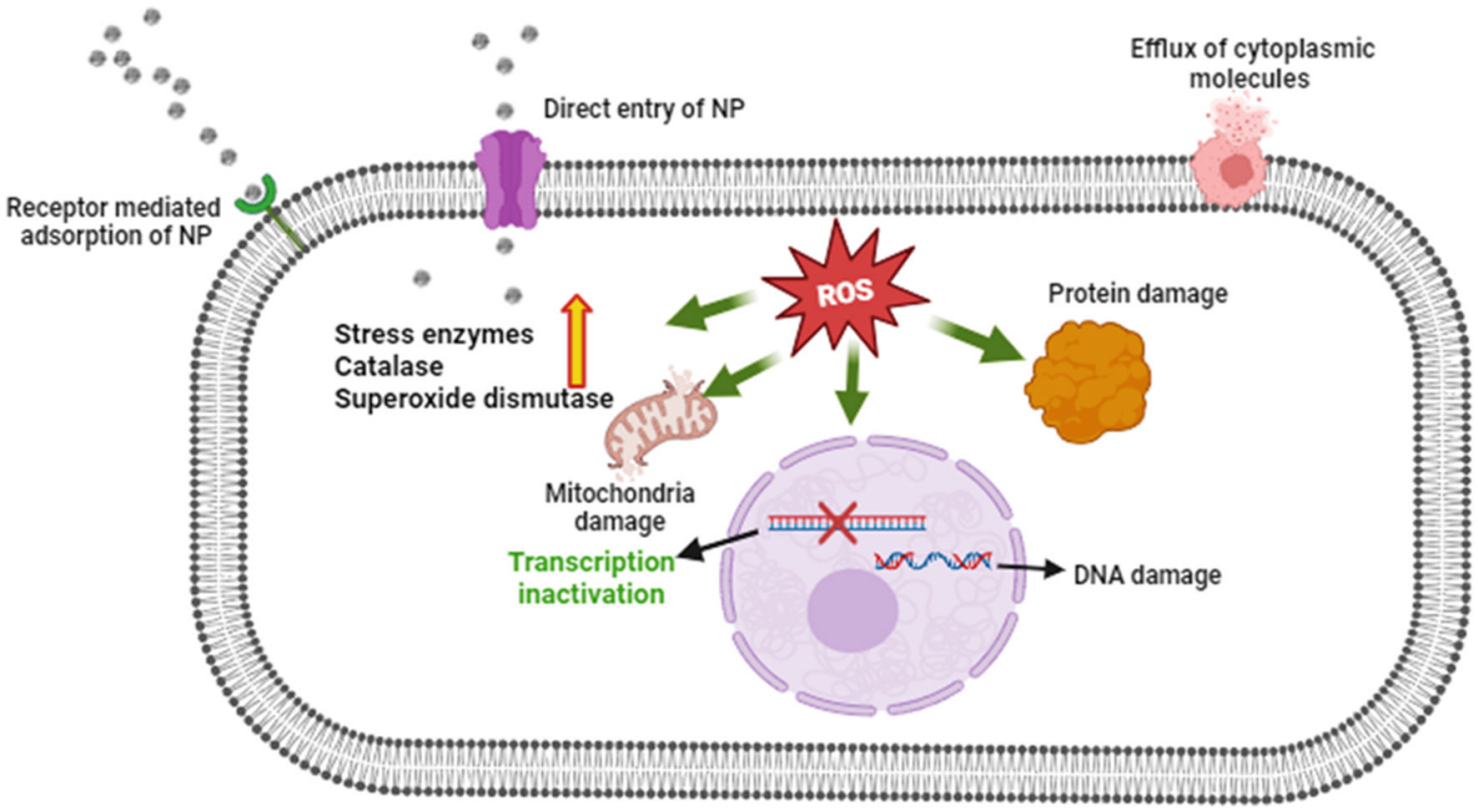
Proposed antifungal mechanism of M-ZnO NPs.

The antifungal potential of M-ZnO NPs and C-ZnO NPs at six concentrations on PDA *in-vitro* was investigated using the mycelial growth inhibition (MGI) assay. Significant MGI observations were recorded on the 12th day of incubation attributed to the slow growth rate of the tested pathogen (Figures 8A, B, Supplementary Table 3). The effectiveness of the M-ZnO NPs and C-ZnO NPs increased in a dose-dependent manner. The percent inhibition of mycelial growth of *A. brassicae* was found to be 38.51 ± 1.55, 44.07 ± 1.527, 50.37 ± 0.52, 62.96 ± 2.04, 78.88 ± 1.1, and 91.48 ± 0.3%, respectively, at concentrations of 10, 25, 50, 100, 150, and 200 μg/ml in M-ZnO NPs supplemented media. Similarly, the media supplemented with C-ZnO NPs at equal concentrations, mycelial growth inhibition (%) was 34.81 ± 2.40, 39.25 ± 1.557, 48.88 ± 2.51, 59.23 ± 2.64, 71.11 ± 1.00, and 79.62 ± 0.55. Compared with the control treatment, the MGI (91.48%) of *A. brassicae* was significantly lower at 200 μg/mL M-ZnO NPs followed by M-ZnO NPs (78.88%) and C-ZnO NPs (79.62%) at 150 and 200 μg/mL, respectively. Like the control, Tween 80 (1.00%) and salt-enriched media (1,000 μg/mL) also had no inhibitory effects, maintaining a percent inhibition of 0.00 ± 0.00%. Compared with the antifungal efficacy of M-ZnO nanoparticles, the fungicide mancozeb was found to be less effective, achieving only 82.96 ± 0.577% mycelial growth inhibition. Among all the treatments, the M-ZnO nanoparticles were the most effective, and inhibited >90% of the radial growth. The antifungal mechanism of ZnO NPs suggested in previous studies that NPs can enter fungal cells *via* a direct and indirect mechanisms, allowing them to access fungal cells *via* direct contact with the cell wall and endocytosis, respectively. Upon entry ROS are generated by ZnO NPs through processes such as Haber-Weiss and Fenton-like reactions. Zinc ions are released from fungal cell walls, leading to oxidation of the primary components, which in turn leads to cell wall breakdown (Figure 9). These ROS trigger oxidative stress, which leads to lipid peroxidation and protein denaturation and disrupts vital cellular functions. Furthermore, this oxidative stress can directly damage DNA, disrupt transcription processes and impair the ability of the cell to repair and replicate (Manke et al., 2013; Fu et al., 2014).

### 3.6 Inhibitory effect on fungal conidia

The *in vitro* findings clearly demonstrated that the antifungal efficacy of M-ZnO nanoparticles increased significantly with increasing dose. The effects of M-ZnO NPs on the length, width, and percentage of damaged conidia of *A. brassicae* were evaluated (Supplementary Table 4). The average length of the conidia in the control sample was 127.99 ± 1.51 μm and the conidial width was 45.66 ± 1.20 μm. At 10, 25, 50, 100, 150, and 200 μg/mL M-ZnO NPs, the conidial length decreased to 120.05 ± 0.33, 113.18 ± 1.53, 106.88 ± 2.22, 102.52 ± 1.16, and 94.55 ± 1.14 μm (*p* value < 0.005), and the conidial width was decreased to 36.33 ± 1.45, 32.66 ± 1.20, 30.33 ± 1.85, 26.23 ± 1.15, and 24.55 ± 0.57 μm (*p* value < 0.005), respectively. Furthermore, the application of 0.2% mancozeb resulted in significant reductions in both the conidial length and width of 98.37 ± 0.93 and 29.33 ± 2.60 μm, respectively.

**Figure 10:**
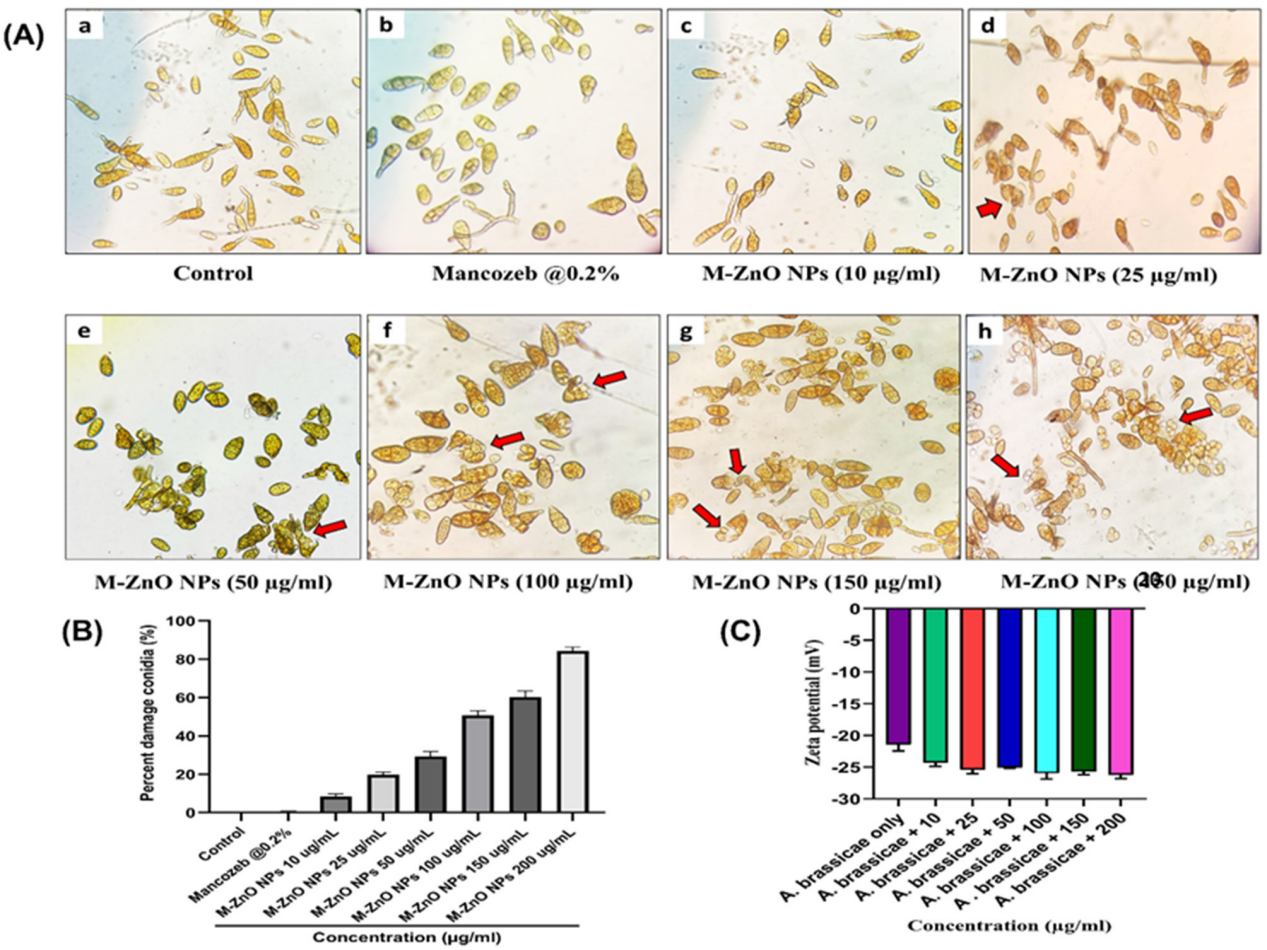
Microscopic and zeta potential analysis of *A. brassicae* treated with M-ZnO NPs: **(A)** Damaged spores of *A. brassicae* treated with various concentrations of M-ZnO under light microscope (40× magnification), **(B)** percent damage conidia treated with various concentrations of M-ZnO NPs, and **(C)** zeta potential of fungal cell treated with different concentrations of M-ZnO NPs. Data represents mean ± standard errors of three replicates by one-way-ANOVA (Tukey, HSD, *p* ≤ 0.05). Lower case letter is the concentraion of nanoparticles namely 10, 25, 50, 100, 150, and 200 μg/ml.

After a 7-day incubation period with different concentrations of M-ZnO NPs, microscopic observations revealed that the conidia of *A. brassicae* were damaged in the treated samples compared with those in the untreated control samples (Figures 10A, B). The control had no effect on spore damage (0%). In contrast, mancozeb at a concentration of 0.2% resulted in minimal spore count damage, which was 0.33%, suggesting that this fungicide has a negligible effect on spore damage. However, for the M-ZnO NPs, a corresponding increase in spore count damage was observed with increasing concentration. The spore damage counts were 8.66 ± 1.20, 19.66 ± 0.88, 31.66 ± 1.45, 49.33 ± 0.88, and 60.33 ± 1.85% at concentrations 10, 25, 50, 100, 150, and 200 μg/ml M-ZnO NPs, respectively. This finding indicates that even low concentrations of M-ZnO NPs can cause some degree of damage to spores. According to Jain et al. (2020), bacterial filtrate based ZnO nanoparticles disrupt hyphal membranes and generate oxidizing agents, which can also affect the integrity of spores.

### 3.7 Determination of zeta potential of fungal hyphae treated with M-ZnO NPs

The cell surface of fungi is usually negatively charged due to the presence of elements such as melanin and negatively charged glycoproteins in the cell wall. This negative charge plays a crucial role in determining the interaction between ZnO nanoparticles and fungal hyphae, which depends on electrostatic bonding (Akpomie et al., 2021). Negative zeta potentials in conidial suspensions are particularly important as they maintain cell dispersion and stability, thereby preventing aggregation. The dispersion ensures effective interactions with nanoparticles as aggregation would reduce the available surface area for binding. To validate the hypothesis that electrostatic interactions influence the attachment of ZnO nanoparticles, the zeta potential of fungal cells with and without M-ZnO NPs was examined. As shown in Figure 10C, fungal cells alone exhibited lower negative charges, whereas fungal cells exposed to different concentrations of M-ZnO NPs exhibited relatively similar negative charges. This finding suggests that positively charged M-ZnO nanoparticles can improve the colloidal stability of fungal cells, possibly by neutralizing or reducing surface charge. The improved colloidal stability facilitates the attachment of M-ZnO nanoparticles to the fungal cell surface, thereby increasing their effectiveness against fungal pathogens.

### 3.8 SEM-EDX analysis of fungal hyphae treated with M-ZnO NPs

**Figure 11:**
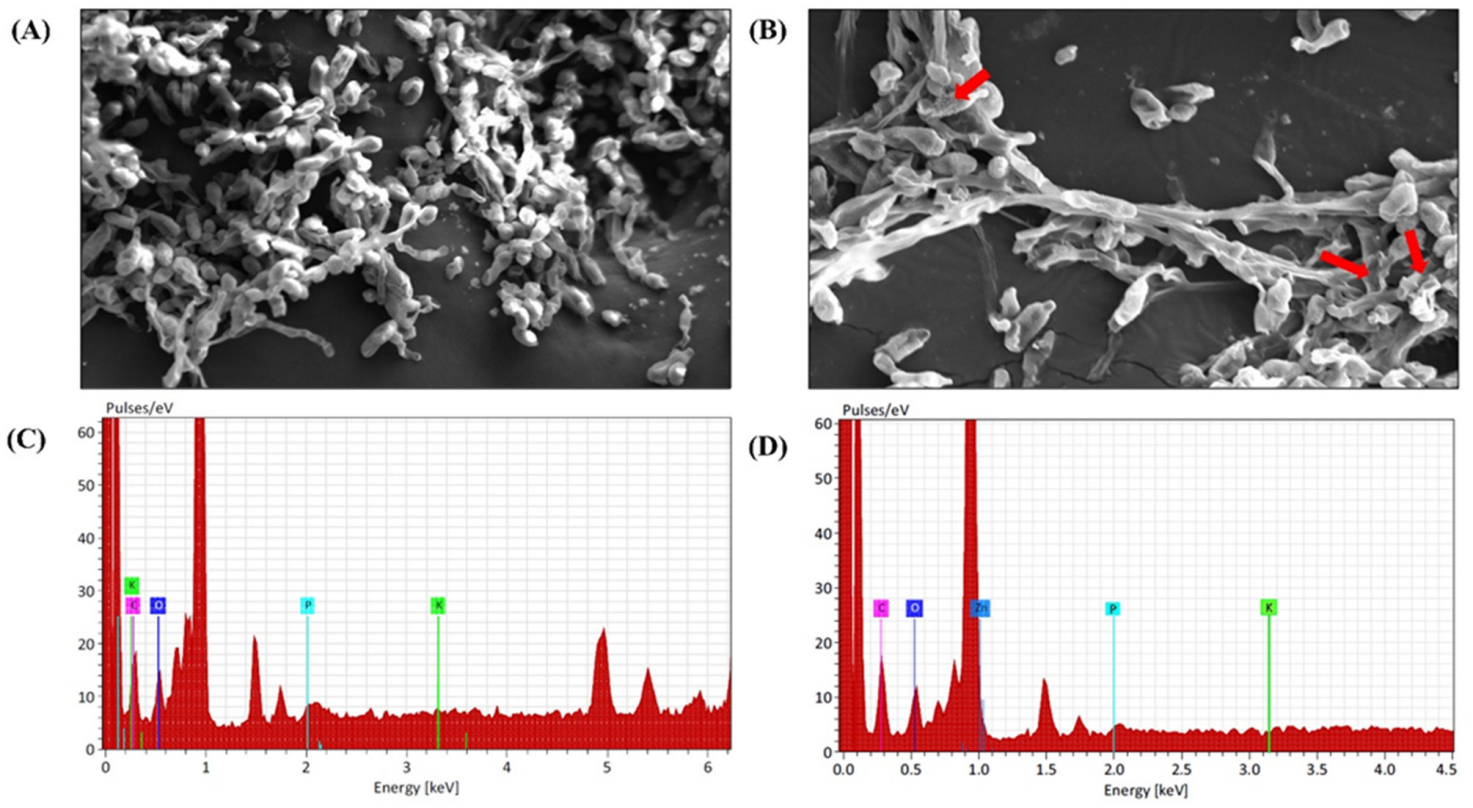
SEM and EDX analysis of *A. brassicae* after treatment with M-ZnO NPs: **(A)** control: without treatment, **(B)** mycelium after treatment with M-ZnO NPS (200 mg/L), **(C)** EDX analysis of control (untreated) mycelium, and **(D)** EDX analysis of treated mycelium (200 mg/L).

Various studies have reported patterns of surface adhesion and uptake when nanoparticles interact with biological samples, including fungi, bacteria, and viruses (He et al., 2011). SEM analysis revealed that the mycelia of *A. brassicae* deformed after exposure to M-ZnO NPs, which was probably due to disturbances in chitin formation and the release of cellular material into the outer environment. Compared with the control, treatment with 200 μg/ml M-ZnO NPs resulted in irregularly shrunken hyphae (Figures 11A, B). In the control, the mycelium appeared to be healthy with smooth and turgid surface. EDX spectra revealed the elements found in the treated and untreated fungal samples (Figures 11C, D). The peaks corresponding to potassium, carbon, oxygen and phosphorus were detected in the control sample. In contrast, the fungal mycelia treated with M-ZnO NPs contained carbon, oxygen, zinc, phosphorus, and potassium. The presence of a zinc peak in the fungal sample suggests the successful attachment of M-ZnO NPs.

### 3.9 M-ZnO NPs induced ROS and reduced plasma membrane viability of *A. brassicae*

**Figure 12:**
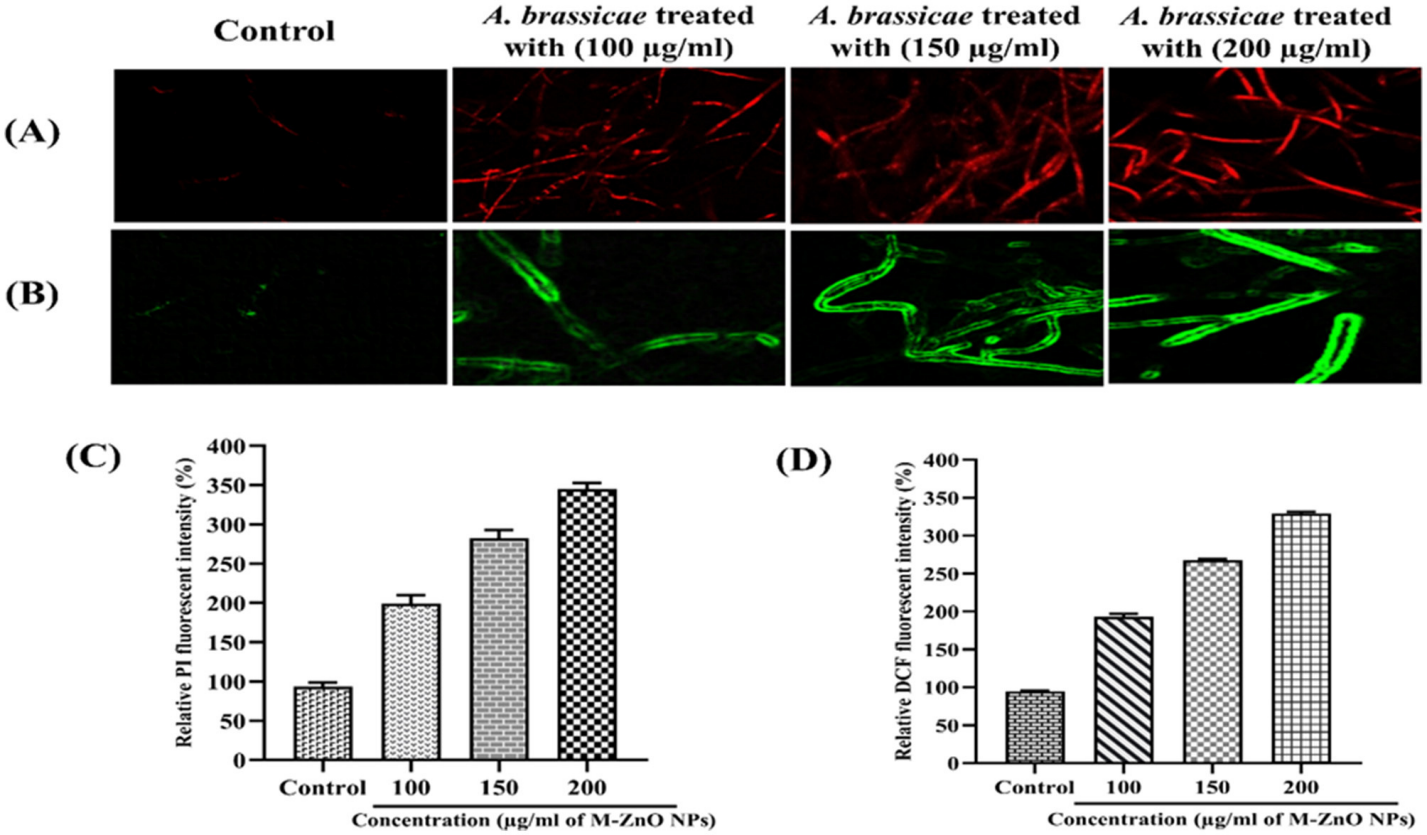
Red fluorescent protein (RFP) images of control (untreated) and treated hyphae **(A)**, Green fluorescent protein images of control (untreated) and treated hyphae **(B)**, and **(C, D)** represents the fluorescent intensity of PI and DCFDA in mycelium treated with M-ZnO NPs. Data represents mean ± standard errors of three replicates by one-way-ANOVA (Tukey, HSD, *p* ≤ 0.05).

The fungicidal properties of the M-ZnO NPs were further confirmed by use of fluorescent dyes such as H2DCFDA and PI. Compared with those of the control hyphae, the fluorescence intensity of the fungal hyphae significantly increased in dose dependent manner. The fluorescent DNA probe PI infiltrates the plasma membrane of a damaged cell, resulting in red fluorescence emanating from the stained cell nucleus (Ahamad Khan et al., 2023). The membrane integrity of M-ZnO NPs treated and untreated *A. brassicae* hyphae is shown in Figures 12A, C. The red fluorescent protein images revealed that the red fluorescence of the treated hyphae was stronger than that of the control hyphae, which only presented slight red fluorescence. After a 20-min exposure time, the PI fluorescence intensities of the treated groups (with 100, 150, and 200 μg/mL M-ZnO NPs) were 2.02, 2.92, and 3.48-fold higher than those of the control group, respectively.

The accumulation of ROS is shown by the green fluorescent protein micrographs of the M-ZnO NPs treated fungal mycelia and the untreated control mycelia (Figures 12B, D). The H2DCFDA fluorescence intensity in *A. brassicae* treated with 100, 150, and 200 μg/mL M-ZnO NPs increased by 1.98, 2.62, and 3.28 times, respectively, compared with that in the untreated control group. Positively charged ZnO nanoparticles exhibit non-specific interactions with negatively charged cell membranes and cause significant cytotoxicity and genotoxicity as a result of enhanced cellular uptake via adsorptive endocytosis. This interaction disrupts membrane permeability (Wingett et al., 2016). The primary mechanism by which ZnO nanoparticles exhibit antimicrobial activity is the production of ROS. Subsequently, cellular homeostasis is disrupted by NADH oxidation and disruption of microbial electron transport chains. These effects collectively contribute to the antifungal properties (Krishnamoorthy et al., 2022).

The results of our study suggest that M-ZnO NPs disrupt the oxidation-reduction balance by producing ROS. This phenomenon might be related to the mechanism of action of M-ZnO NPs, which involves the formation of pores in the cell membrane and the transport of NPs in *A. brassicae* cells. Previous studies have documented how ZnO NPs produce ROS, emphasizing the role of the Fenton reaction and the Haber-Weiss cycle. The above comparison of fluorescence intensities suggested that M-ZnO NPs treatment enhanced fungus-nano interactions, resulting in a greater variation in the free energy content and ultimately an increase in ROS generation.

### 3.10 Nanoparticles induced stress in *A. brassicae*

The oxidative stress occurs in fungal cells due to the frequent generation of ROS by internal or external stress. To balance this ROS, fungi evolved antioxidant mechanisms that include enzyme families e.g., superoxide dismutase (SOD), peroxidases and catalases (CAT), (Pradhan et al., 2021). The first protective enzyme to emerge during oxidative stress is SOD, which facilitates the production of H_2_O_2_ in response to extracellular stress. The enzyme catalase is responsible for scavenging H_2_O_2_. For this reason, these two important antioxidant enzymes are essential for fungal stress resistance.

**Figure 13:**
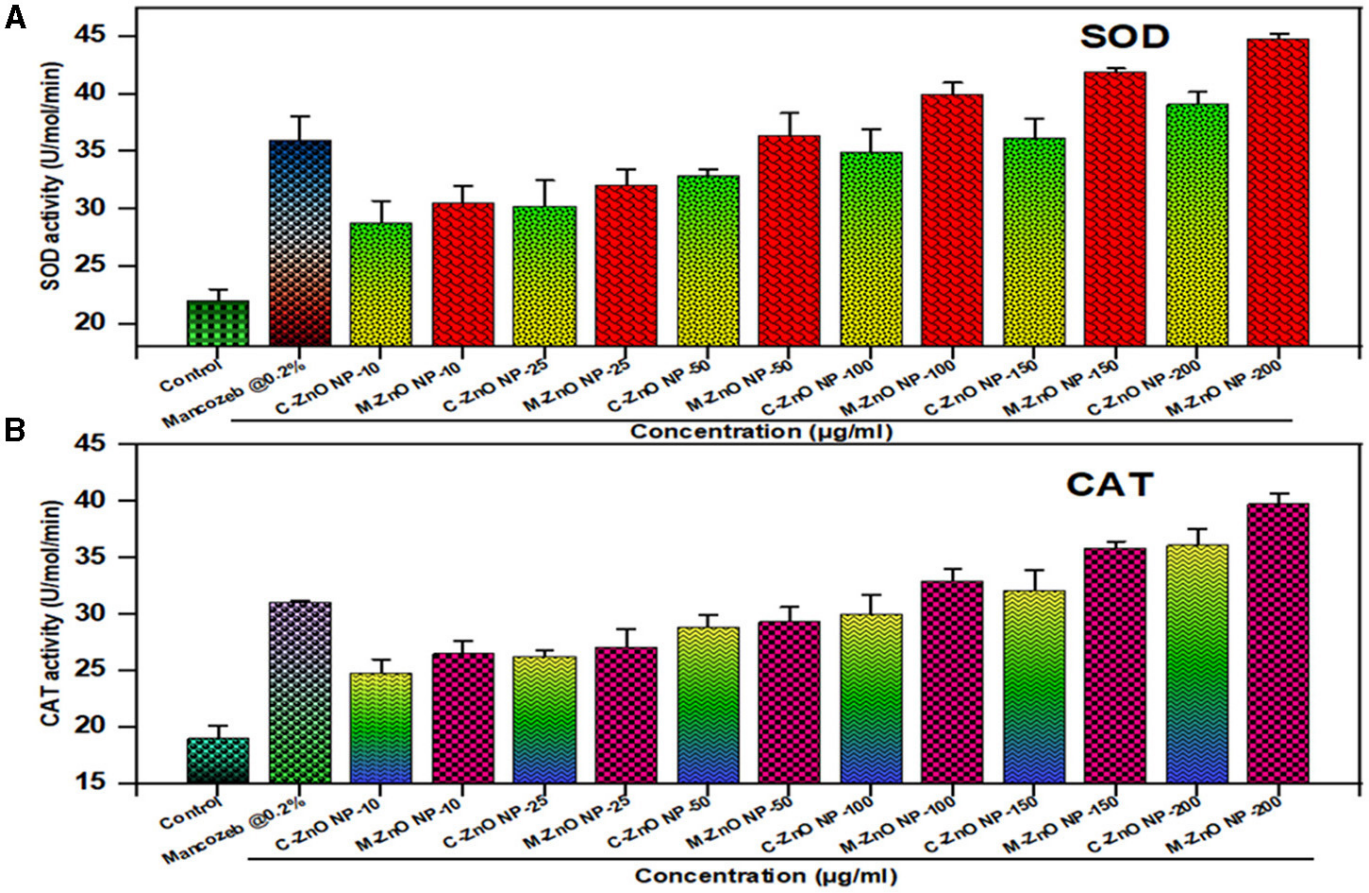
Effect of different treatments on defense enzyme activity in *A. brassicae*, **(A)** superoxide dismutase (SOD), and **(B)** catalase (CAT). Data represents mean ± standard errors of three replicates by one-way-ANOVA (Tukey, HSD, *p* ≤ 0.05).

In our study (Supplementary Table 5), we found that stressed fungal cells treated with M-ZnO NPs presented a maximum SOD level of 44.2 U/mol, whereas those treated with C-ZnO NPs presented a SOD level of 39.1 U/mol and a basal ROS level in the control (untreated; Figure 13A). Dose-dependent activity of SOD was observed at sublethal concentrations, while an almost twofold increase was recorded at the maximum concentration (200 ppm). Similarly, M-ZnO NPs treated fungal cells presented an increase in CAT activity of approximately 39.6 U/ml, whereas the activity of C-ZnO NPs was 36.4 U/mol (Figure 13B). Although the application of mancozeb at a concentration of 0.2% led to increase in CAT and SOD of 31 and 36 U/mol, respectively, it was less effective than the M-ZnO NPs. Thus, the maximum increase in oxidative stress is clearly because of the catalytic activity of the M-ZnO NPs.

## 4 Discussion

Nanoparticles have gained substantial attention in agriculture as new-generation fungicides due to their ability to overcome traditional fungicide resistance, environmentally friendly and provide sustained antifungal activity. Previous studies have documented the use of various fungal species such as *Fusarium, Penicillium* and *Trichoderma* for the synthesis of nanoparticles (Hassan et al., 2019; Badawy et al., 2021; Shaheen et al., 2021). In the present study *T. harzianum* was used as a reducing and stabilizing agent for the mycosynthesis of ZnO NPs because it secretes various antifungal compounds. The CFCF of *T. harzianum* facilitated the reduction of Zn^2+^ to ZnO nanoparticles through the production of bioactive compounds such as enzymes and secondary metabolites. These biomolecules acted as capping agents and ensured the stability and dispersion of the synthesized nanoparticles (Consolo et al., 2020; Zaki et al., 2021).

To optimize the mycosynthesis process, various physicochemical parameters *viz.*, CFCF, substrate concentration, pH and temperature, were determined through systematic screening. These physicochemical properties of the synthesized nanoparticles and their UV-Vis absorption peak between 200 and 400 nm, are consistent with previous findings (Sumanth et al., 2020; Zaki et al., 2021), suggesting the importance of these physicochemical factors for the mycosynthesis of ZnO NPs.

The concentration of CFCF played an essential role in the reaction. At 10 mL, the optimal absorbance peak was observed at 362 nm, indicating successful nanoparticle synthesis. Deviations in the filtrate concentration led to the aggregation of nanoparticles. Similar results were also reported by Shobha et al. (2020), who reported that the size and characteristics of ZnO NPs are dependent on the concentration of Trichoderma culture filtrate. Similarly, different concentration of the substrate solutions was examined but the characteristic absorption peak was found at 359 nm with a concentration of 5 mM. Similar work was pursued by Al-Rasheedi et al. (2022), who found that the biosynthesis of zinc oxide nanoparticles is affected by the concentration of the salt substrate. These results suggest that the synthesis of ZnO NPs is very sensitive to such parameters, and changes affecting nanoparticle properties, including absorbance and particle size. The pH of the reaction medium affects nucleation and stability of the ZnO nanoparticles. The optimal result was observed at pH 9, indicated by an absorption peak at 350 nm. At this alkaline pH, hydroxyl ions facilitate complete reduction and stabilization, thus preventing the aggregation observed at lower pH (6–7). Deviations from this pH disrupt the capping of the nanoparticles, resulting in irregular morphologies and reduced uniformity. Ashraf et al. (2015) and Gherbi et al. (2022) concluded that larger particles typically form at very low and high pH values, such as pH values of 6 and 10, which affects their application in different fields. Temperature has significant effects on the kinetics of the nanoparticle synthesis process. The temperature of 70°C was optimal for the synthesis of ZnO NPs, with an absorption peak at 360 nm. Temperatures below this range failed to provide sufficient energy for the complete reduction of Zn^2+^ ions, while excessively high temperatures (>70°C) destabilized the reaction mixture and lead to particle aggregation. The same approach was used by Mohammadi and Ghasemi (2018) and Chandra and Gopchandran (2021), who reported that the size of ZnO nanoparticles depends on the temperature of the solution.

Structural characterization of the synthesized M-ZnO nanoparticles revealed their crystal structure and morphology. The XRD analysis showed distinct diffraction peaks corresponding to the hexagonal wurtzite crystal structure of ZnO NPs. The diffraction peaks at 2θ angles corresponding to (100), (002), and (101) planes were indexed using the JCPDS card No. 01-075-1526 (Jamdagni et al., 2018; Thein et al., 2017) and the size of the M-ZnO NPs was 29 nm.

DLS analysis revealed a hydrodynamic particle size of 50.79 nm, which is slightly larger than the crystallite size due to the hydration layers surrounding the particles. The polydispersity index (PDI) showed a moderately uniform size distribution. Zeta potential measurements revealed a negative surface charge of −17.49 mV, which leads to electrostatic repulsion between the particles (Makarov et al., 2014). This electrostatic repulsion improves colloidal stability and reduces aggregation tendencies (Kim et al., 2014).

FTIR analysis confirmed the synthesis of M-ZnO NPs with a characteristic Zn-O stretching peak at 552 cm^−1^. Functional groups such as C=O (1,056, 1,248 cm^−1^), N-O (1,510 cm^−1^) and C=C (1,658, 2,860 cm^−1^) were present on the surface of M-ZnO NPs, which facilitate reduction of metal ions and stabilization of the ZnO nanoparticles. The O-H stretching bands (3,726 cm^−1^) contributed to the stability of nanoparticles and prevented agglomeration (Contreras-Cornejo et al., 2016; Shobha et al., 2020; Waris et al., 2021; Yao et al., 2023; Herrera Pérez et al., 2024).

TGA analysis of M-ZnO NPs showed an initial weight loss below 150°C due to moisture and volatile compounds, followed by a significant loss between 200 and 400°C due to the decomposition of organic capping agents and biomolecules. Beyond 400°C, the weight stabilized, confirming the high thermal stability and the role of *T. harzianum* metabolites in nanoparticles synthesis. Our results are consistent with findings from other studies (Fatimah et al., 2016; Quadri et al., 2017; Sagadevan et al., 2019).

SEM analysis showed a combination of spherical, rod and hexagonal shapes of M-ZnO NPs with a size range of 20–50 nm and uniform distribution. HR-TEM confirmed their crystalline nature with a size range of 10–40 nm, lattice fringes (*d-*spacing ~0.26 nm), and polycrystalline structure from SAED patterns. HR-TEM confirmed lattice fringes that were in line with the XRD results, indicating a consistent crystallite structure (Geetha et al., 2016). Our findings align with the results of Zaki et al. (2021), who reported that coagulation processes are the reason for the skeletal structure of ZnO nanoparticles. The sizes of the particles were consistent with the results of XRD and DLS, which further confirmed the reliability of the synthesis process.

EDX analysis confirmed a near-stoichiometric composition of M-ZnO NPs with 73.15% zinc and 26.85% oxygen, indicating successful synthesis and high purity. The absence of impurities highlights the effectiveness of the green synthesis method. Shnawa et al. (2022), who also conducted an elemental analysis of ZnO nanoparticles and reported that the nanoparticles have a high degree of purity, with approximately 78.5% zinc and 21.5% oxygen.

M-ZnO NPs showed superior antifungal activity against *A. brassicae*, with 91.48% mycelial growth inhibition at 200 μg/mL compared to C-ZnO NPs (79.62%) and mancozeb (82.96%). The nanoparticles showed a dose-dependent inhibitory effect, with significant reductions in mycelial growth and spore viability. At the highest concentration, M-ZnO NPs caused 60.33% spore damage and significant deformation of fungal hyphae, which was further confirmed by SEM-EDX analysis. Various studies have shown that ZnO NPs obtained from different fungal culture filtrates have antifungal activity in the range of 100–300 μg/ml (Miri et al., 2019; Vargas Hernández et al., 2024; Abdelaziz et al., 2022). Our results suggest that M-ZnO NPs are able to kill fungal pathogens even at lower concentrations as they have high antifungal potential at 150–200 μg/mL. M-ZnO NPs also effectively inhibited the conidial development of *A. brassicae* by reducing both the length and width of conidia in a dose-dependent manner. The highest reduction was observed at 200 μg/mL (M-ZnO NPs), the average conidial length decreased from 127.99 ± 1.51 μm (control) to 94.55 ± 1.14 μm and the width reduced from 45.66 ± 1.20 μm to 24.55 ± 0.57 μm. These results are consistent with the findings of Dhiman et al. (2022) on the antifungal effectiveness of ZnO NPs synthesized from *Terminalia bellerica*. These nanoparticles have a relatively large surface area, so they can easily adhere to and be absorbed by the conidia surface, resulting in the collapse of the spore structure (He et al., 2011). These nanomaterials can serve as a reliable means to assess their toxic effects on fungi (Angelé-Martínez et al., 2017).

Zeta potential analysis revealed that fungal hyphae treated with M-ZnO NPs had increased negative surface charge compared to untreated cells, indicating stronger electrostatic interactions. This shift increases colloidal stability and facilitates the adhesion of nanoparticles to fungal surfaces, contributing to their antifungal activity. According to Miyake et al. (1990), reduced electrostatic repulsion is responsible for the increased adhesion of antifungal drugs to pathogens. Therefore, nanoparticles might be able to damage fungal hyphae, which was further confirmed by SEM analysis. SEM-EDX analysis revealed that fungal mycelia treated with 200 μg/mL M-ZnO NPs were deformed and exhibited irregularly shrunken hyphae, which may be due to the disruption of chitin formation and the release of cell contents. The presence of zinc in treated samples confirmed the attachment of M-ZnO NPs to fungal hyphae, align with the results reported by Xue et al. (2014) who demonstrated similar uptake patterns of ZnO NPs using EDX analysis.

The antifungal activity of M-ZnO NPs resulted in significant ROS generation and membrane damage in *A. brassicae* hyphae. ROS levels increased in a dose-dependent manner, with fluorescence intensity at 200 μg/mL (M-ZnO NPs) found 3.28 times higher than the control. Membrane integrity analysis using PI staining showed significant damage at 200 μg/mL (M-ZnO NPs), with fluorescence intensity 3.48 times higher in treated hyphae compared to untreated samples. These results suggest that M-ZnO NPs disrupt cellular homeostasis through oxidative stress, leading to lipid peroxidation and loss of membrane integrity, ultimately affecting fungal viability (Amaldoss et al., 2022). The induction of ROS in *A. brassicae* by M-ZnO NPs, confirming ROS-mediated cell damage as the key antifungal mode of action.

The defense enzyme activity revealed that 200 μg/mL (M-ZnO NPs), had the maximum SOD activity (44.2 U/mol) and CAT activity (39.6 U/mol), compared to C-ZnO NPs (SOD: 39.1 U/mol, CAT: 36.4 U/mol) and mancozeb (SOD: 31 U/mol, CAT: 36 U/mol). This dose-dependent increase demonstrates the ability of M-ZnO NPs to generate ROS, which leads to oxidative stress in fungal cells, disrupt cellular homeostasis and increase antifungal efficacy. Our results are consistent with previous studies highlighting the role of antioxidant enzymes in mitigating ROS-induced damage during oxidative stress (Bin and Feng, 2018; Dhiman et al., 2022).

## 5 Conclusion

The aim of this study was to develop and investigate the antifungal activity of mycosynthesized ZnO nanoparticles against *A. brassicae*. The M-ZnO NPs showed the highest antifungal activity against *A. brassicae* at 200 μg/mL with 91.48% mycelial growth inhibition compared to C-ZnO NPs and mancozeb (0.2%). Light microscopic examination revealed that the maximum reduction in spore length and width (94.55 ± 1.14 and 24.55 ± 0.57 μm) was achieved with M-ZnO NPs at 200 μg/mL. Furthermore, maximum spore damage percentage (60.33 ± 1.85%) was also observed for M-ZnO NPs at 200 μg/mL. SEM-EDX analysis showed the deformation in fungal mycelium and the absorption of M-ZnO NPs. Furthermore, a confocal laser scanning microscopy study indicated generation of cytoplasmic ROS and membrane damage in *A. brassicae* cells supplemented with M-ZnO NPs. Fungal mycelia treated with M-ZnO NPs showed maximum enzymatic stress activity compared to C-ZnO NPs and mancozeb (0.2%). M-ZnO NPs showed remarkable antifungal activity against *A. brassicae*. This suggests that M-ZnO NPs have the potential to be a sustainable and effective alternative to traditional fungicides for controlling fungal diseases in plants.

## Data Availability

The original contributions presented in the study are included in the article/Supplementary material, further inquiries can be directed to the corresponding authors.
