# Supplementary material for: Synthesis of zinc oxide nanoparticles using Trichoderma harzianum and its bio-efficacy on Alternaria brassicae
Source: Front Microbiol. 2025 Feb 13;16:1506695. doi: 10.3389/fmicb.2025.1506695 (PMC11864937; doi:10.3389/fmicb.2025.1506695)
Supplement: Supplementary file 1 [file Table_1.DOCX]

**Supplementary Table 1:** Peak position, d-spacing value, and miller indices for M-ZnO NPs

| **Sr. No.** | **Peak position (2*θ*)** | **d-spacing value (Å)** | **Miller indices (*hkl*)** | **FWHM (Radians)** | **Crystallite size (nm)** |
| --- | --- | --- | --- | --- | --- |
| 1 | 31.778 | 2.813629 | 100 | 0.26 | 29.38757 |
| 2 | 34.445 | 2.601634 | 2 | 0.266 | 28.52663 |
| 3 | 36.253 | 2.475925 | 101 | 0.306 | 24.6733 |
| 4 | 47.509 | 1.912275 | 102 | 0.232 | 31.34158 |
| 5 | 56.56 | 1.625857 | 110 | 0.21 | 33.31467 |

**Supplementary Table 2:** FTIR analysis of M-ZnO NPs and *Trichoderma harzianum* filtrate

| **FTIR Spectra of M-ZnO NPs** | | **FTIR Spectra of Control (*T. harzianum*)** | |
| --- | --- | --- | --- |
| **Peaks (cm^-1^)** | **Types of bonds** | **Peaks (cm^-1^)** | **Types of bonds** |
| 552 | Zn-O | - | - |
| 1056 | C=O | 1049 | CO-O-CO |
| 1248 | C=O | 1119 | C=O |
| 1510 | N-O | 1514 | N-O |
| 1658 | C=C | 1662 | C=C |
| 2860 | N-H | 2875 | N-H |
| 3340 | N-H | 3310 | N-H |
| 3624 | O-H | 3549 | O-H |
| 3726 | O-H | 3668 | O-H |

**Supplementary Table 3:** Efficacy of M-ZnO NPs against *A. brassicae* under *in vitro* conditions

| **Sr No.** | **Treatments** | **Radial growth (mm)*** | **Percent Inhibition (%)*** |
| --- | --- | --- | --- |
| 1 | Control | 90.00 | 0 (0.000 ± 0.000^)#l^ |
| 2 | Tween 80 1000@ μg ml^−1^ | 90.00 | 0 (0.000 ± 0.000)^l^ |
| 3 | Salt @200 μg ml^−1^ | 90.00 | 0 (0.000 ± 0.000)^l^ |
| 4 | Mancozeb @0.2% | 15.33 | 82.960 (65.621±0.577)^b^ |
| 5 | M-ZnO NPs 10@ μg ml^−1^ | 55.33 | 38.512 (38.362±1.552)^j^ |
| 6 | M-ZnO NPs 25@ μg ml^−1^ | 50.33 | 44.077 (41.592±1.529)^i^ |
| 7 | M-ZnO NPs 50@ μg ml^−1^ | 44.67 | 50.374 (45.212±0.525)^g^ |
| 8 | M-ZnO NPs 100@ μg ml^−1^ | 33.33 | 62.961 (52.513±2.044)^e^ |
| 9 | M-ZnO NPs 150@ μg ml^−1^ | 19.00 | 78.881 (62.647±1.100)^c^ |
| 10 | M-ZnO NPs 200@ μg ml^−1^ | 7.67 | 91.482 (73.03 ± 0.321)^a^ |
| 11 | C- ZnO NPs 10@ μg ml^−1^ | 58.67 | 34.815 (36.159 ± 2.044)^k^ |
| 12 | C- ZnO NPs 25@ μg ml^−1^ | 54.67 | 39.254 (38.795 ±1.522)^j^ |
| 13 | C- ZnO NPs 50@ μg ml^−1^ | 46.00 | 48.885 (44.360 ± 2.512)^h^ |
| 14 | C- ZnO NPs 100@ μg ml^−1^ | 36.00 | 59.230 (50.765 ± 2.645)^f^ |
| 15 | C- ZnO NPs 150@ μg ml^−1^ | 26.00 | 71.110 (57.485 ±1.000)^d^ |
| 16 | C- ZnO NPs 200@ μg ml^−1^ | 18.33 | 79.620 (63.170 ± 0.552)^c^ |

*Data are the mean of three replications. #Data within parentheses are Angular-transformed values. Value ± Standard errors followed by different letters in each column indicate a significant difference (Tukey HSD, *p*≤0.05). M-ZnO NPs: mycogenic zinc oxide nanoparticles, C-ZnO NPs: Chemically synthesized zinc oxide nanoparticles.

**Supplementary Table 4:** Average dimensions and damage percentage of conidia after treatment with M-ZnO NPs

| **Sr. No.** | **Treatments** | **Conidial length (µm)** | **Conidial width (µm)** | **Percent conidia damage(%)** |
| --- | --- | --- | --- | --- |
| 1 | Control | 127.99±1.51^a^ | 45.66±1.20^a^ | 0±0^g^ |
| 2 | Mancozeb@0.2% | 98.37±0.93^ef^ | 29.33±2.60^bcd^ | 0.33±0.333^g^ |
| 3 | M-ZnO NPs 10µg/ml | 120.05±0.33^b^ | 36.33±1.45^b^ | 8.66±1.20^f^ |
| 4 | M-ZnO NPs 25 µg/ml | 113.18±1.53^c^ | 32.66±1.20^bc^ | 19.66±0.88^e^ |
| 5 | M-ZnO NPs 50 µg/ml | 106.88±2.22^d^ | 30.33±1.85^bcd^ | 31.66±1.45^d^ |
| 6 | M-ZnO NPs 100 µg/ml | 102.52±1.16^de^ | 26.23±1.15^cd^ | 49.33±0.88^c^ |
| 7 | M-ZnO NPs 150 µg/ml | 94.55±1.14^f^ | 24.55±0.57^d^ | 60.33±1.85^b^ |
| 8 | M-ZnO NPs 200 µg/ml | 93.35±0.83^f^ | 22.66±0.88^d^ | 82.33±1.15^a^ |

Data are the mean of three replications. Value ± Standard errors followed by different letters in each column indicate a significant difference (Tukey HSD, *p*≤0.05).

**Supplementary Table 5:** Defense enzyme activity in *A. brassicae* treated with M-ZnO NPs and other treatments

| **Sr No.** | **Treatment** | **CAT (U/mol)** | **SOD (U/mol)** |
| --- | --- | --- | --- |
| 1 | Control | 19.22±1.10^h^ | 22.52±0.84^i^ |
| 2 | Mancozeb @0.2% | 31.02±0.33^d^ | 36.30±0.22^d^ |
| 3 | C-ZnO NP-10 | 24.77±0.55^g^ | 28.81±1.18^h^ |
| 4 | C-ZnO NP-25 | 26.22±2.33^fg^ | 30.25±2.1^g^ |
| 5 | C-ZnO NP-50 | 28.68±0.88^ef^ | 32.88±1.01^f^ |
| 6 | C-ZnO NP-100 | 29.96±1.55^de^ | 34.66±1.76^e^ |
| 7 | C-ZnO NP-150 | 32.11±0.33^cd^ | 36.10±1.77^d^ |
| 8 | C-ZnO NP-200 | 36.40±1.86^b^ | 39.10±2.4^c^ |
| 9 | M-ZnO NP-10 | 26.55±2.15^fg^ | 30.51±2.1^g^ |
| 10 | M-ZnO NP-25 | 27.05±1.66^ef^ | 32.12±1.94^f^ |
| 11 | M-ZnO NP-50 | 29.35±0.56^de^ | 36.40±1.26^d^ |
| 12 | M-ZnO NP-100 | 32.92±1.1^cd^ | 39.96±1.05^bc^ |
| 13 | M-ZnO NP-150 | 35.90±0.90^bc^ | 41.75±0.55^b^ |
| 14 | M-ZnO NP-200 | 39.60±0.33^a^ | 44.20±0.88^a^ |

Data are the mean of three replications. Value ± Standard errors followed by different letters in each column indicate a significant difference (Tukey HSD, *p*≤0.05).
